# Comprehensive Genomic Characterization and Probiotic Properties Evaluation of *Limosilactobacillus fermentum* JAC 231 Isolated from Vaginal Microbiota

**DOI:** 10.1007/s12602-026-11025-7

**Published:** 2026-04-28

**Authors:** Carmem Duarte Lima Campos, Amanda Graziella Goncalves Mendes, José Lima Pereira-Filho, Viviane da Silva Sousa Almeida, Ana Beatriz Santos Sousa, Israel Viegas Moreira, João Lucas do Carmo Lima, Aleania Polassa Almeida Pereira, Allysson Kayron Carvalho Silva, Laís Araújo Souza Wolff, Kátia Regina Assunção Borges, Cristianne Roberta Rhoden, Cinara Regina Aragão Vieira Monteiro, Cristina de Andrade Monteiro, Elizabeth Soares Fernandes, Valério Monteiro-Neto

**Affiliations:** 1 Centro de Ciências Biológicas e da Saúde, Programa de Pós-Graduação em Ciências da Saúde – PPGCS, Universidade Federal do Maranhão, Avenida dos Portugueses, 1966, campus Bacanga, São Luís, MA 65080-040 Brazil; 2 Programa de Doutorado em Biotecnologia – Rede Nordeste de Biotecnologia (RENORBIO), Universidade Federal do Maranhão, Avenida dos Portugueses, 1966, campus Bacanga, São Luís, MA 65080-040 Brazil; 3 Laboratório Cedro, Avenida Silva Maia, 81, Centro, São Luís, MA 65020-570 Brazil; 4 Present Address: Instituto Federal de Educação Ciência e Tecnologia do Maranhão, Avenida Getúlio Vargas, 4, Monte Castelo, São Luís, MA 65030-005 Brazil; 5 Department of Animal and Veterinary Sciences - ANIVET Ruminant Nutrition (RUN), Aarhus University, Viborg, Denmark

**Keywords:** *Limosilactobacillus fermentum*, Probiotic, Vaginal microbiota, Whole-genome sequencing, Antimicrobial activity, Mucosal adhesion, Safety assessment

## Abstract

*Limosilactobacillus fermentum* JAC 231, a strain isolated from the vaginal microbiota of an asymptomatic woman, was investigated for its genomic features, safety, probiotic potential, and antimicrobial properties in this study. Whole-genome sequencing revealed a 2.06 Mbp genome with 51.4% GC content and 1,993 predicted coding sequences (CDS). Functional annotation indicated that the genes were related to carbohydrate metabolism, stress tolerance, adhesion, and bacteriocin biosynthesis. Comparative analysis revealed that *L. fermentum* JAC 231 shared 1,587 orthologous groups with the other five probiotic strains of *L. fermentum*. No virulence factors or transferable antibiotic resistance genes were detected, and only an incomplete prophage region was identified, which supports the safety of this strain. Phenotypically, JAC 231 exhibited tolerance to acidic pH and bile salts and demonstrated high adhesion capacity to porcine gastric mucin (5.97 log₁₀ CFU/mL) and HeLa epithelial cells (5.79 log₁₀ CFU/mL). Antimicrobial susceptibility testing indicated resistance to vancomycin and moderate susceptibility to ciprofloxacin, features considered intrinsic and nontransferable. The strain exhibited broad-spectrum antimicrobial activity, with inhibition zones ranging from 16.60 ± 2.08 mm to 28.30 ± 2.88 mm against various Gram-positive and Gram-negative pathogens. Agar diffusion assays indicated that the antimicrobial activity of the cell-free supernatant was primarily due to the production of organic acids. Furthermore, JAC 231 significantly reduced pathogen adherence to epithelial cells in competitive, exclusion, and displacement assays. Collectively, these genomic and functional attributes support the safety and probiotic potential of *L. fermentum* JAC 231 and underscore its promise as a candidate for therapeutic applications targeting the urogenital and gastrointestinal microbiota.

**Supplementary Information:**

The online version contains supplementary material available at 10.1007/s12602-026-11025-7.

## Introduction

In 2020, Zheng and colleagues performed a comprehensive taxonomic revision of the genus *Lactobacillus*, which resulted in the creation of several new genera and the reclassification of *Lactobacillus fermentum* as *Limosilactobacillus fermentum* [1]. This species is classified within the lactic acid bacteria (LAB) group, comprising Gram-positive, catalase-negative, acid-tolerant, nonsporulating, and strictly fermentative microorganisms [2]. Strains of *L. fermentum* are highly versatile and inhabit a wide variety of ecological niches, including fermented foods, plant materials, and the gastrointestinal and urogenital tracts of humans [3–7].

In addition to their well-established technological applications in the food industry, particularly in enhancing food quality and shelf life [8, 9], several *L. fermentum* strains have shown promising probiotic attributes. Probiotics are defined as “live microorganisms that, when administered in adequate amounts, confer a health benefit on the host” [8]. Notably, *L. fermentum* strains exhibit various beneficial activities, including reinforcement of epithelial barrier function, modulation of host immune responses [4, 9], competitive exclusion of pathogens [10, 11], and production of antimicrobial substances such as organic acids, hydrogen peroxide, and bacteriocins with a broad spectrum of activity [12–14]. These properties confirm previous investigations into the therapeutic potential of *L. fermentum* strains against disorders affecting the gastrointestinal and vaginal tracts [15–17].

Recent comparative genomic analyses have also highlighted the probiotic potential of *L. fermentum* strains isolated from the human oral cavity, such as *L. fermentum* SD7, which displays aggregation-related genes, absence of virulence and resistance determinants, and strong phylogenetic coherence within distinct clades, underscoring its candidacy for food and healthcare applications [18].

The efficacy and safety of probiotics are strain-specific, necessitating the rigorous evaluation of individual strains rather than extrapolating data across species [8]. Hence, the thorough characterization of probiotic candidates involves not only the demonstration of beneficial physiological effects but also comprehensive safety assessments, such as confirming the absence of antibiotic resistance transferability and virulence factors, and testing the strain’s tolerance to gastrointestinal stresses, such as acidic pH, digestive enzymes, and bile salts [8, 19, 20]. With advances in genome sequencing technologies, genomic analyses have emerged as essential tools for detailed characterization, providing insights into the genetic features related to the safety, metabolic capabilities, and probiotic potential of individual strains [21–24]. Some genomic studies on various probiotic *Lactobacillus* species have underscored the importance of genome-level characterization for reliably predicting probiotic functions and safety in humans [24–27].

In a previous study by our group, novel probiotic candidates were isolated from the vaginal microbiota of healthy women without symptoms of genital infection. Among these isolates, *L. fermentum* JAC 231 demonstrated significant antifungal and antivirulence effects against *Candida albicans*, including the reduction of hydrolytic enzyme secretion, inhibition of hyphal formation, impairment of coaggregation and adhesion to epithelial cells, and disruption of biofilm development [28]. Building on these promising results, the present study aimed to sequence and comprehensively analyze the genome of *L. fermentum* JAC 231, evaluate its safety profile and tolerance under simulated gastrointestinal conditions, and further characterize its antimicrobial activity and ability to inhibit the adherence of bacterial pathogens to eukaryotic host cells.

## Materials and Methods

### Bacterial Strains and Growth Conditions

The microorganisms used in this study were the *L. fermentum* JAC 231 probiotic strain isolated from the vaginal microbiota of healthy women and strains acquired from the American Type Culture Collection (ATCC, Manassas, VA): *L fermentum* ATCC 23271), *Klebsiella pneumoniae* ATCC 700063, *Pseudomonas aeruginosa* ATCC 27853, *Escherichia coli* (ATCC 25922), *Staphylococcus aureus* ATCC 25923, *Salmonella enteritidis* ATCC 13076, *Enterococcus faecalis* (ATCC 29212), and enteropathogenic *Escherichia coli* O127:H6 E2348/69 (EPEC) from the Basic and Applied Microbiology Laboratory of the Federal University of Maranhão.

*L. fermentum* JAC 231 and ATCC 23,271 strains were grown De Man, Rogosa, and Sharpe (MRS) agar and broth (Difco Laboratories, Detroit, MI, USA) under anaerobic conditions, while the other bacteria were grown in broth and brain heart infusion (BHI) agar under aerobic conditions. All microorganisms were stored at −80 °C until use.

### Sequencing of the Complete Genome of *L. fermentum* JAC 231

Genome sequencing of *L. fermentum* JAC 231 was performed using Neoprospecta Microbiome Technologies genomic services (Florianópolis, SC, Brazil). Genomic DNA was extracted using the PureLink^®^ Genomic DNA Mini Kit (Invitrogen, USA) following the manufacturer’s instructions. The complete genome was sequenced using the Illumina MiSeq paired-end library approach and prepared using the Nextera XT DNA Library Preparation Kit (Illumina, San Diego, CA, USA), resulting in an estimated genome coverage of approximately 35–50×. Raw Illumina reads (FASTQ format) were used for de novo genome assembly analysis. No long-read sequencing technologies (such as Oxford Nanopore or PacBio) were used. Raw reads exhibited high quality, with a mean Phred score of 30.76 and 71.24% of bases presenting Phred scores ≥ Q30. The OneShotWGS Bacteria pipeline was used to assemble the genome. This pipeline uses A5 [29], which features assembly corrections using the ID-BA-UD [30] and Spades programs [31]. To identify a potential mixing profile of organisms in a sample, the 16 S and 23 S rRNA ribosomal genes were taxonomically assigned using BLASTN algorithm [32]. Protein prediction was performed from the assembled genome using the Prokka program [33].

### Genome Analysis

The genome sequence of *L. fermentum* JAC 231 underwent comprehensive annotation and functional analysis using Rapid Annotation using Subsystem Technology (RASTtk v. 1.3.0) pipeline [34, 35] and KEGG database [36]. Protein similarity searches against the NCBI databases were performed using BLAST. To ensure methodological consistency, all strains included in the analysis (*L. fermentum* MCC 2760, AGR 1485, ATCC 23271, CECT 5716, and IFO 3956) were re-annotated using the Prokka pipeline [33] prior to their comparison. Orthologous proteins among these strains were analyzed using OrthoVenn 3 [37]. Phylogenetic analyses were performed using nucleotide sequences from the Type (Strain) Genome Server (TYGS) [38, 39]. The genomic content was visualized using the CGView server [40].

Mobile genetic elements, including plasmids and bacteriophages, were identified using PlasmidFinder v2.1 and PHASTER [41], and CRISPR arrays and Cas proteins were analyzed using CRISPRCasFinder v. 1.1.2 [42]. Pathogenicity and virulence determinants were assessed using PathogenFinder v.1.1 [43] and VirulenceFinder v.2.0 [44]. Antimicrobial resistance genes were detected using ResFinder v.4.0 [45], RAST [34], and CARD v3.2.4 [46]. Bacteriocin-coding genes were investigated using BAGEL5 [47] and manually confirmed using BLASTp 2.17.0 [48].

### Safety and Probiotic Properties of *L. fermentum* JAC 231

*L. fermentum* ATCC 23271 served as the probiotic positive control in all following assays due to its known probiotic characteristics (Itapary Dos Santos et al. 2019).

#### Antibiotic Susceptibility Testing

Antibiotic susceptibility was evaluated using disk diffusion as described by Charteris et al. [49] and the broth microdilution method according to the CLSI guideline [50]. For the disc diffusion method, antibiotics tested (Oxoid/Thermo Fisher Scientific, Basingstoke, UK) included chloramphenicol (30 µg), tetracycline (30 µg), norfloxacin (10 µg), rifampicin (5 µg), penicillin (10 µg), erythromycin (15 µg), nitrofurantoin (300 µg), gentamicin (120 µg), cefazolin (30 µg), tobramycin (10 µg), clindamycin (2 µg), vancomycin (30 µg), sulfazotrim (25 µg), oxacillin (1 µg), novobiocin (5 µg), ciprofloxacin (5 µg), teicoplanin (30 µg), tigecycline (15 µg), cefoxitin (30 µg), and linezolid (30 µg). Plates were incubated anaerobically at 37 °C for 24 h, and the inhibition zones were measured in mm.

The Minimum Inhibitory Concentration (MIC) of *L. fermentum* JAC 231 against vancomycin (0.031–64 µg/ml). was determined using 96-well microtiter plates. Inocula were prepared from colonies grown for 24 h at 37 °C under anaerobic conditions and standardized using a spectrophotometer (OD600 nm = 0.1). The antibiotic was serially diluted across the wells. The standardized bacterial suspension was then inoculated into the wells and incubated anaerobically at 37 °C for 48 h. MICs were recorded as the lowest concentration of vancomycin at which visible bacterial growth was inhibited.

#### Tolerance to Gastrointestinal Conditions

Resistance to acidic pH and bile salts (Oxgall, Sigma–Aldrich) was assessed as described by Monteiro et al. [51]. Briefly, aliquots of MRS broth adjusted to pH 2 or 4 or supplemented with 5 g/L or 10 g/L bile salts were inoculated with 100 µL of a 24-hour culture and incubated anaerobically for 3 h at 37 °C. The control consisted of MRS broth without bile salts or was adjusted to pH 7. After incubation, the samples were serially diluted, plated on MRS agar, and incubated at 37 °C for 24 h. The percentage of viable cells relative to the control was calculated from colony counts obtained on MRS agar using the following formula: Survival rate (%) = (CFU test condition/CFU control) × 100.

#### Mucin Binding Assay

Mucin adhesion capacity was tested using porcine gastric mucin type III (Sigma–Aldrich, Lyon, France), as described by Carmo et al. [10]. Briefly, 100 µL of a 10 mg/mL solution of Porcine Gastric Mucin Type III (Sigma, Saint-Quentin-Fallavier, France) was solubilized in PBS (pH 7.2), added to 24-well microtiter plates, and allowed to bind to the wells for 16 h at 4 °C. The wells were washed with PBS and saturated with 2% (w/v) bovine serum albumin (BSA) solution (Sigma-Aldrich, St. Louis, MO, USA) for 4 h at 4 °C and washed again. with PBS. The bacterial inocula in MRS broth were washed with PBS and standardized using a spectrophotometer (OD600 nm = 0.1). Aliquots of 500 µL of the bacterial suspension were added to each well, and the microplates were incubated at 37 °C for 1 h. The wells were then washed thrice with 1 mL PBS to remove non-adherent bacteria. The wells were treated with 500 µL of 0.5% Triton X-100 (Sigma-Aldrich), and the plates were incubated for 30 min at room temperature with orbital shaking. To quantify the number of bacteria capable of binding mucin, the wells were scraped, serially diluted, plated on MRS agar, and incubated at 37 °C for 24 h under anaerobic conditions. Wells containing mucin without bacteria were used as negative controls.

#### Adhesion to HeLa Cells

Adherence to human cervical epithelial (HeLa) cells was evaluated as described by Carmo et al. [10]. Briefly, human cervical epithelial (HeLa) cells were cultured in Dulbecco’s modified Eagle’s medium (DMEM) containing GlutaMAX (Gibco, USA) supplemented with 1× antibiotic-antimycotic solution (Gibco, USA) and 10% fetal bovine serum (Gibco, USA) at 37 °C under 5% CO_2_. HeLa cells (5 × 10^5^ cells/mL) were cultured in 24-well plates and incubated at 37 °C in 5% CO_2_ atmosphere. Before the adhesion assay, *Lactobacillus* cultures were grown in MRS agar and standardized (5 × 10^8^ CFU/mL) using DMEM. After 24 h, the cells, which had grown to approximately 70% confluence, were washed with PBS, treated with 250 µL of the standard *L. fermentum* JAC 231 suspension plus 250 µL of DMEM, and incubated for 4 h at 37 °C in 5% CO_2_. The wells were then washed and treated with 500 µL of 0.1% Triton X-100 (Sigma, USA) for 5 min while shaking. To quantify the number of adherent cells, serial dilutions and counts were performed on MRS agar plates. The results are expressed as Log10 CFU/mL. HeLa cells incubated in the absence of bacteria were used as negative controls. To visualize bacterial adherence to HeLa cells, the cells were fixed with methanol (Amresco, Gymea, Australia) and stained using a rapid panotype kit. The coverslips were mounted on glass slides and visualized using an optical microscope (100× magnification).

#### Interference with Adhesion to HeLa Cells

Competition, exclusion, and displacement assays were performed to evaluate the effect of *L. fermentum* JAC 231 on the adhesion of pathogenic bacteria to HeLa cell monolayers. After preparing the standard suspensions, the tests proceeded as follows: (I) in the competition assay, *L. fermentum* JAC 231 and the pathogens were inoculated simultaneously with HeLa cells and incubated for 4 h at 37 °C; (II) in the exclusion assay, *L. fermentum* JAC 231 was inoculated and incubated initially for 1 h at 37 °C with HeLa cells, after which the pathogens were added and incubated for another 3 h; (III) in the displacement assay, pathogens were added and incubated for 1 h at 37 °C, after which *L. fermentum* JAC 231 was added and incubated for an additional 3 h. Serial dilutions and plating were performed to quantify the number of adherent cells in each sample. The results are expressed as Log10 CFU/mL.

#### Antagonism Assay

Antagonism assays were performed using the overlay technique, as described by Chew et al. [52]. Cultures of *L. fermentum* JAC 231 grown in MRS broth for 24 h at 37 °C under anaerobic conditions were standardized (1 × 10^8^ cells/mL), and 10 µL aliquots were deposited in MRS agar medium and incubated for 48 h at 37 °C under anaerobic conditions. After this period, Müeller-Hinton agar medium (Difco Laboratories, Detroit, MI, USA) was added at 50 °C to form a 2 mm thick layer. After solidification, standardized pathogenic bacterial inocula (1 × 10^8^ cells/mL) were prepared, including. *K. pneumoniae* ATCC 700063, *P. aeruginosa* ATCC 27853, *E. coli* ATCC 25922, *S. aureus* ATCC 25923, *S. enteritidis* ATCC 13076, *E. faecalis* ATCC 29212, and enteropathogenic *E. coli* O127:H6 E2348/69 (EPEC). The plates were incubated at 37 °C for 24 h under aerobic conditions, and the inhibition zones were measured (in mm).

#### Antimicrobial Activity of Cell-Free Supernatant

To evaluate whether the antimicrobial activity of the cell-free supernatant (CFS) was attributable to organic acids or proteinaceous substances, *L. fermentum* JAC 231 was cultured in 200 mL of MRS broth for 18 h at 37 °C under anaerobic conditions, following a protocol described by Scillato et al., [53] and Wang and Zang [54]. After incubation, the cultures were centrifuged at 5,000 × g for 15 min at 4 °C, and the supernatant was sterilized by filtration through a 0.22 μm syringe filter (Merk, Millipore, Darmstadt, Germany). The obtained CFS was adjusted to pH 6.0 using 10 M NaOH and subsequently treated with trypsin and proteinase K at a final concentration of 1 mg/mL each, at pH 7.5, for 2 h at 37 °C. The enzymes were then inactivated by heating at 100 °C for 5 min, and the pH was readjusted to the initial value. MRS broth adjusted to pH 6.5 and 4.0 was used as the experimental control.

Following these treatments, the antimicrobial activity of the CFS was evaluated using an agar diffusion assay. Inocula of the pathogenic bacteria used in the antagonism assay were seeded onto Mueller–Hinton agar plates. Wells were created using sterile 1 mL micropipette tips and filled with 100 µL of each treated or untreated CFS. Plates were incubated at 35 °C for 24 h, and antimicrobial activity was assessed by measuring inhibition zones (mm).

### Ethical Aspects

The vaginal isolate *L. fermentum* JAC 231 was obtained from a previous study and approved by the Ethics Committee of CEUMA University (n.° 2.519.446/2018/CEP-UNICEUMA).

### Statistical Analysis

All statistical analyses were conducted using GraphPad Prism software (version 9.0; GraphPad Software, San Diego, CA, USA). Data normality was assessed using the Shapiro–Wilk test. For inhibition assays, adhesion to HeLa cells and mucin, the unpaired Student’s t-test was applied. Two-way ANOVA was used for tolerance tests of gastrointestinal conditions and interference with adhesion to HeLa cells, followed by Dunnett’s test. Statistical significance was set at *P* < 0.05. All assays were performed in triplicate on three separate days.

### Complete Genome Sequence Data Accession Number

This whole-genome shotgun project for *L. fermentum* JAC231 was deposited in the DDBJ/ENA/GenBank under accession number JAVFHY000000000. Raw sequencing reads (FASTQ files) are available in the NCBI Sequence Read Archive (SRA) under the accession number SRR37146884. The BioProject accession number PRJNA1007394 and BioSample is SAMN37068468.

## Results

### Genome Analysis

#### General Characteristics of the *L. fermentum* JAC 231 Genome

**Fig. 1:**
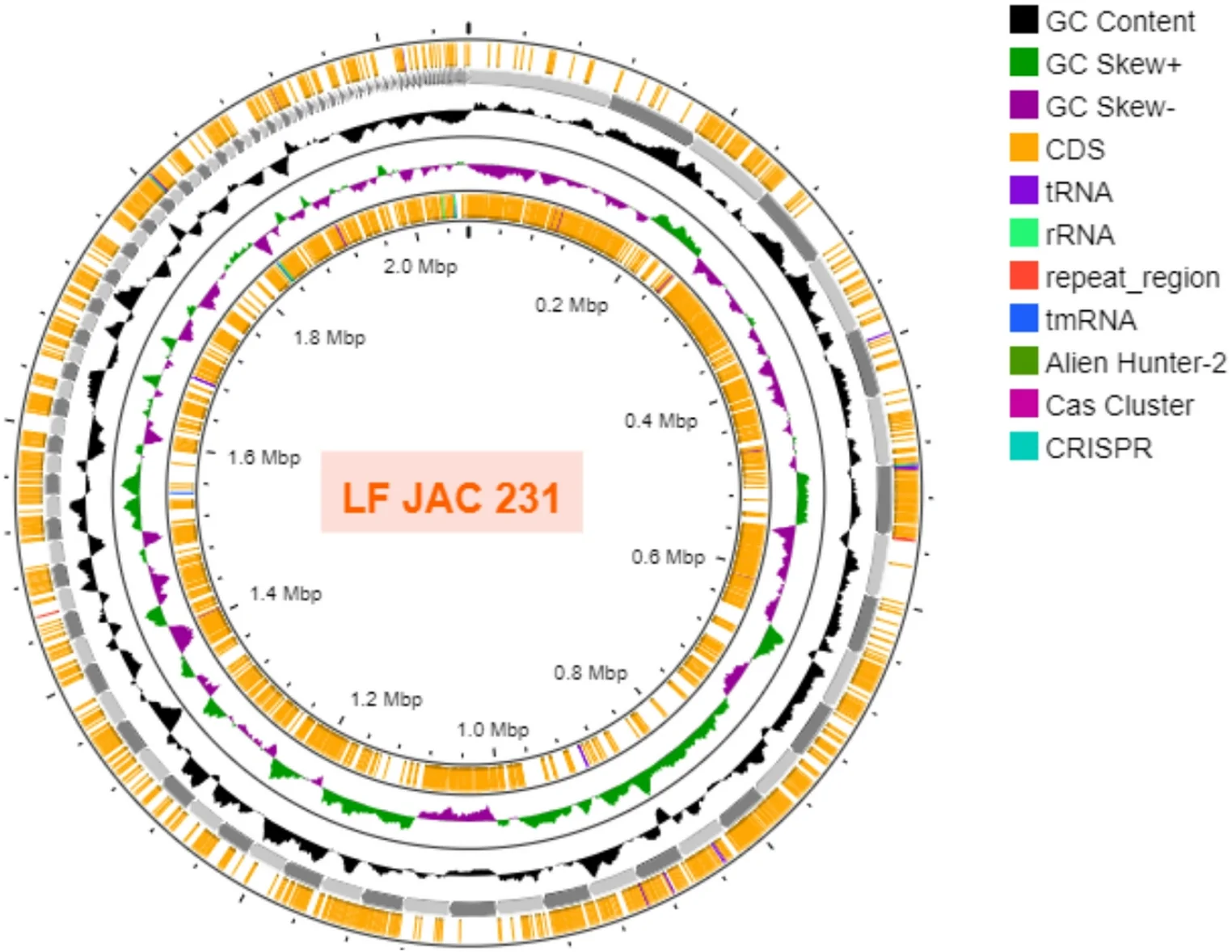
Circular map of the *L. fermentum* JAC 231 genome. The first and last circles represent genes on the positive and negative strands, respectively, including coding sequences (CDS), transfer tRNA, rRNA, and other genes; the third circle represents the GC content; the fourth circle represents the GC slope, where green indicates GC > 0, purple indicates GC < 0, and the junction of green and purple is the starting point and endpoint of replication, respectively

To elucidate the genetic basis of the probiotic properties, the genome of the vaginal clinical strain of *L. fermentum* JAC 231 was sequenced in this study. Table 1 summarizes the general genomic features obtained using RAST. A genome map was generated using CGVIEW (Fig. 1).

**Table 1:** General features of the genome assembly of *L. fermentum* JAC 231

Attribute	Value
Size of genome (bp)	2,064,918
GC (%)	51.4
N50	36,970
L50	20
No of Contigs	133
No of Subsystems	292
No of Coding Sequences	1993
No of RNAs	62
No of operons de tmRNA	1
No of tRNA	55
No of rRNA	6
No of tmRNA	1
No of CRISPR loci	2

**Fig. 2:**
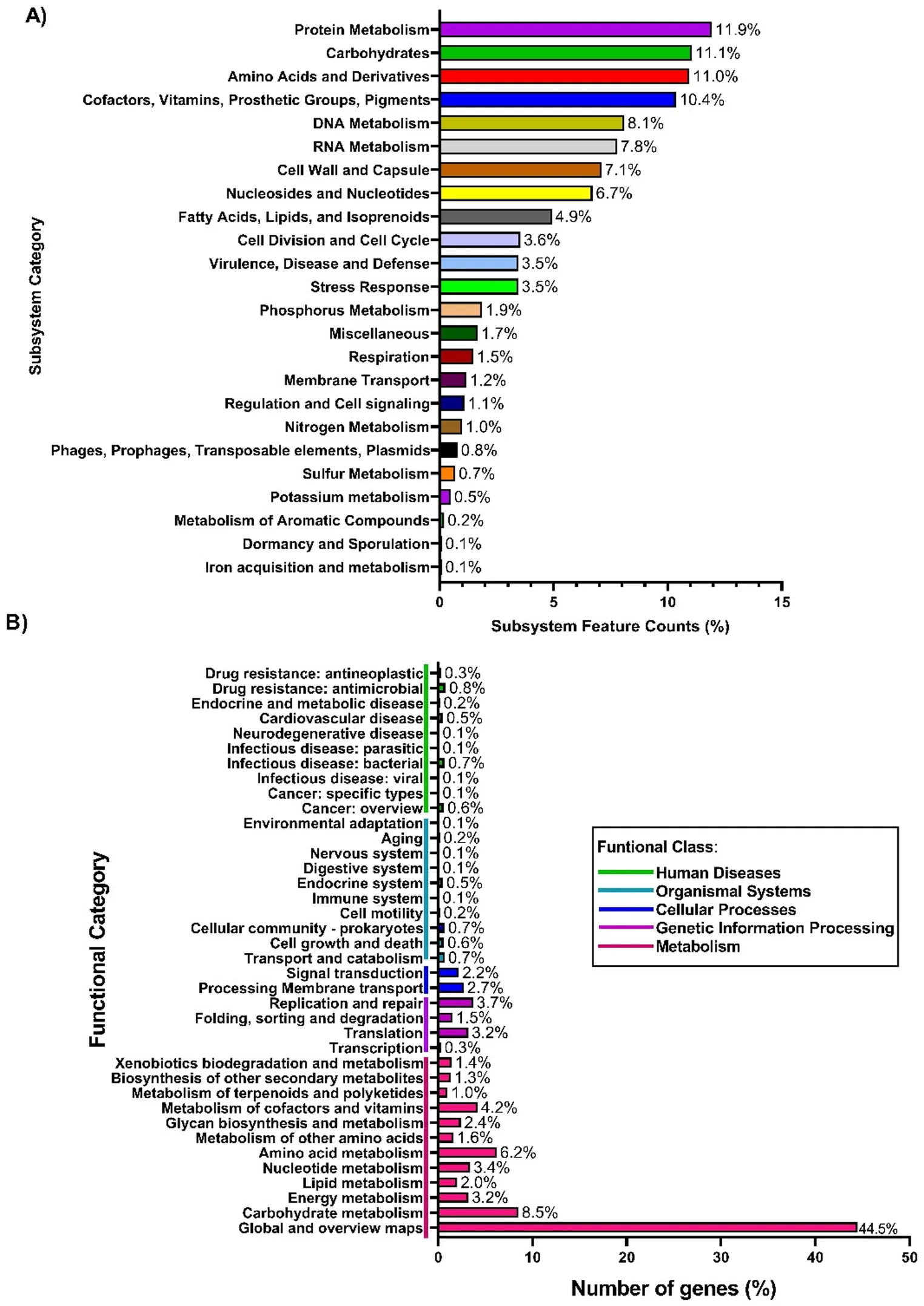
(**A**) Overview of the categories and subsystems of the *L. fermentum* JAC 231 genome annotated using the RAST. (**B**) Kyoto Encyclopedia of Genes and Genomes (KEGG) functional annotation

Genetic prediction performed by RAST revealed the presence of 1,993 protein-coding sequences distributed across 292 subsystems (Table 1). Of these, 1,358 were related to non-hypothetical proteins and 635 to hypothetical proteins. By analyzing the distribution of categories in the subsystems, it was found that the subsystems with the greatest number of genes were related to the metabolism of proteins, carbohydrates, amino acids (and derivatives), cofactors, vitamins, prosthetic groups, pigments, and DNA (Fig. 2A). Functional annotation of the genome was also performed using the KEGG database, where 1,801 genes (56.2%) were annotated and divided into six classes of metabolic pathways (Fig. 2B). The categories with the highest gene abundance were global and overview maps (44.5%), carbohydrate metabolism (8.5%), and amino acid metabolism (6.2%). A comprehensive summary of the identified subsystems and functional resources is presented in Tables S1 (RAST) and S2 (KEGG).

#### Phylogenetic Analysis

**Fig. 3:**
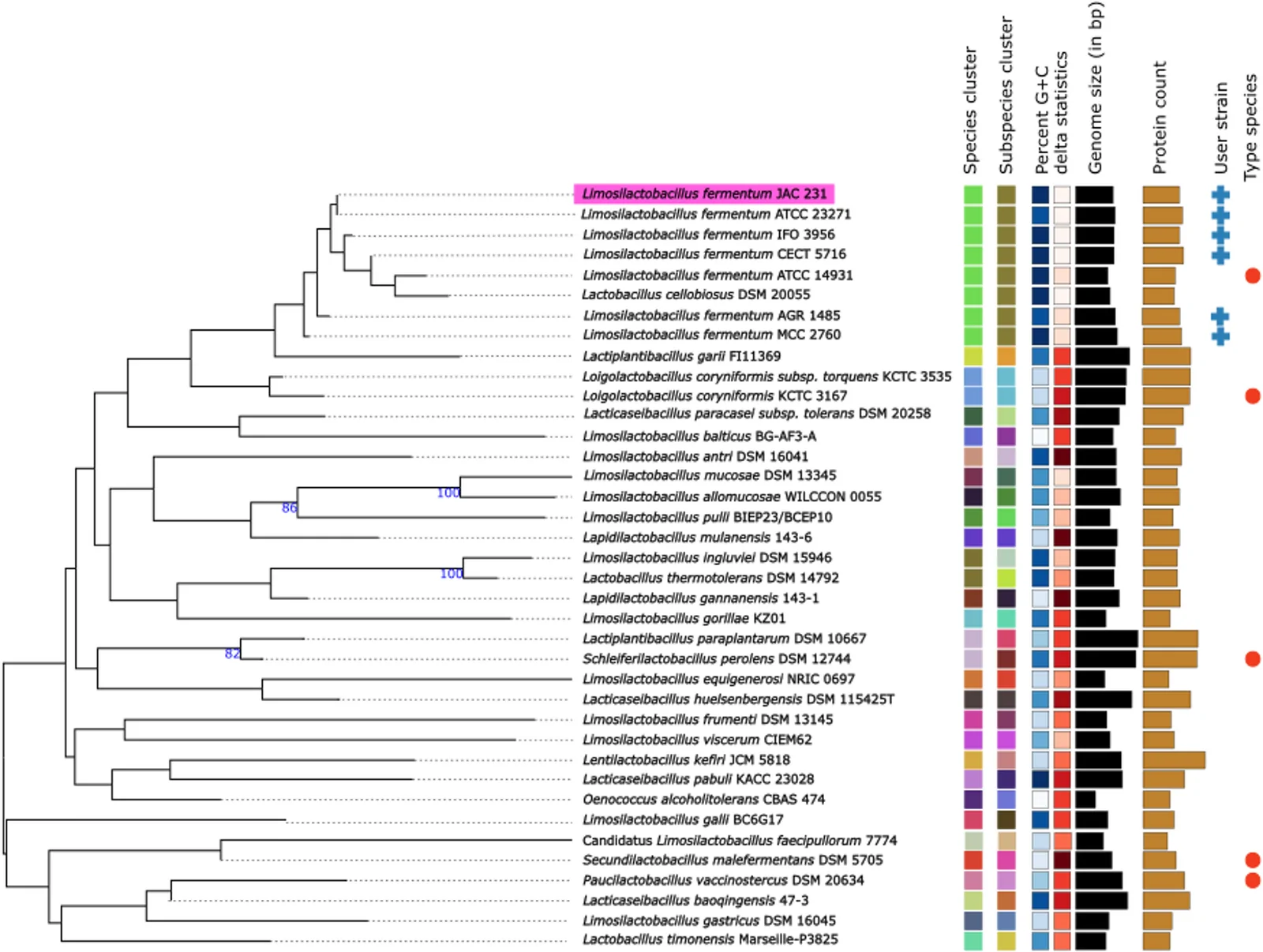
Phylogenetic tree of *Limosilactobacillus fermentum* strain JAC 231 and related taxa. The tree was inferred with FastME 2.1.6.1 [55] from Genome BLAST Distance Phylogeny (GBDP) distances calculated from genome sequences available in the TYGS database. Branch lengths are scaled in terms of GBDP distance formula d5. Numbers above the branches indicate GBDP pseudo-bootstrap support values > 60% from 100 replicates (average branch support: 22.8%). The tree was rooted at the midpoint [56]. Strain JAC 231 is highlighted in magenta and marked with a blue star. Type strain genomes are indicated by red circles. Additional metadata (species, subspecies, experimental source, genome size, and protein count) are shown

A phylogenetic tree was constructed using reference genomes and other sequences available in the TYGS database showing the evolutionary relationships between strains of the genus *Limosilactobacillus* and related taxa (Fig. 3). The clade formed by *L. fermentum* exhibited a confidence level of 100%. *L. fermentum* JAC231 clustered within the *L. fermentum* clade, presenting high bootstrap support values (> 87), which confirm the robustness of its position. Its closest lineage was *L. fermentum* ATCC 23271. *L. cellobiosus* DSM 20055 was positioned within the clade containing JAC231 and other *L. fermentum* strains recognized as probiotics, including AGR 1485, CECT 5716, MCC 2760, and IFO 3956. Comparative visualization demonstrated that JAC 231 shares genomic traits consistent with those of other *L. fermentum* strains, reinforcing its identity and functional potential within the genus.

#### Orthologous cluster analysis

**Fig. 4:**
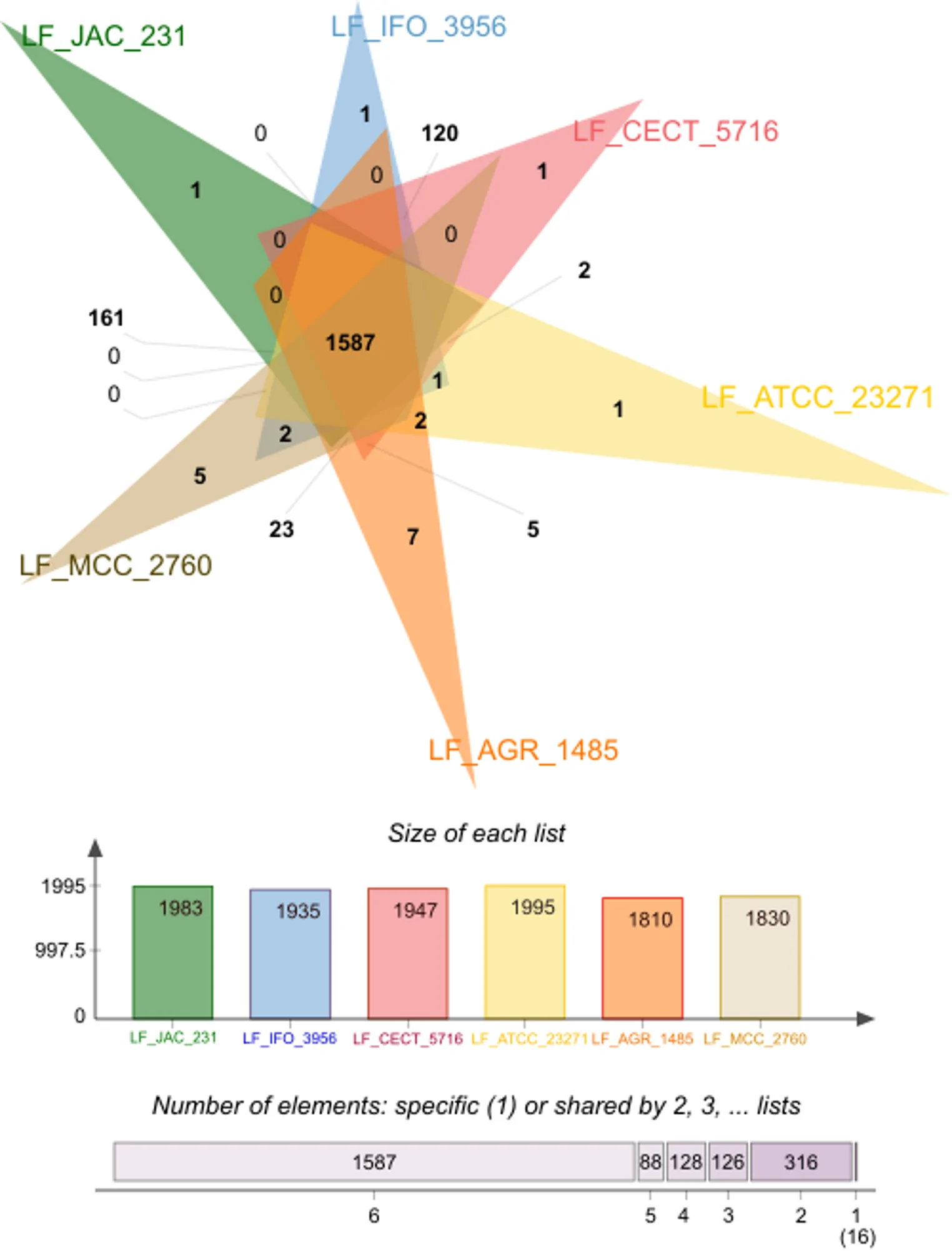
Venn diagram showing the distribution of orthologous clusters shared among the following *L. fermentum* strains: JAC 231, MCC 2760, AGR 1485, ATCC 23271, CECT 5716, and IFO 3956. The Venn diagram represents the number of unique and shared orthologous clusters, whereas the bar graph represents the number of clusters in each strain

Orthologous cluster analysis using OrthoVenn3 (Fig. 4) revealed that the strains formed 2,261 clusters, with 42 overlaps and 1,450 single-copy clusters, encoding 12,902 proteins and 459 singletons (3.56%) (no orthologs). Of these, 1,587 were orthologous clusters common to all strains examined, reflecting essential and conserved genetic features of the species. A supplementary analysis of the distribution of orthologous clusters among *L. fermentum* strains is presented in Figure S1 and S2, highlighting the core genome and strain-specific adaptations of *L. fermentum* JAC 231.

OrthoVenn3 analysis revealed that *L. fermentum* JAC 231 comprises 1,983 orthologous (Figure S2) clusters and a lineage-specific cluster (cluster 2261) containing two proteins unique to *L. fermentum* JAC 231 (Figure S1). Although a Swiss-Prot hit (P75980) was detected, no Gene Ontology annotations were available, and both proteins were classified as hypothetical proteins. These findings indicate that *L. fermentum* JAC 231 harbors unique genes with currently uncharacterized functions that may reflect strain-specific genomic adaptations.

The 1,587 shared clusters included 10,049 proteins, with each strain encoding approximately 16% of these proteins (Fig. S1). Most proteins were involved in biological, metabolic, and cellular processes (Table S3). Furthermore, we observed the presence of orthologous clusters among lineages important for probiotic activity, such as those related to vitamin synthesis, biofilms, fermentation, carbohydrate metabolism, and responses to external stimuli. Regarding molecular functions, 14.5% of the identified proteins were involved in transport activities, and 15.6% were related to molecular and oxidoreductase functions. The third group of proteins was related to cellular and membrane components (Table S3).

#### Analysis of Probiotic Characteristics

We analyzed the probiotic characteristics of the strains through genome annotation using the RAST SEED server, as described above. The following genes involved in vitamin synthesis were screened: thiamine (vitamin B1), riboflavin (vitamin B2), pyridoxine (vitamin B6), biotin (vitamin B7), and folate (vitamin B9) (Table S4). The analysis also revealed the presence of five genes related to adhesion, four related to the biosynthesis of exopolysaccharides (EPSs), and sixty-one involved in the response to oxidative, osmotic, and thermal stress, tolerance to bile salts, and pH. (Table 2).

**Table 2:** Subsystems related to probiotic characteristics identified in the genome of *L. fermentum* JAC 231

Gene Function RAST/BLAST	Gene (Abbrev.)	Query Length	Accession Length	Query Cover (%)	E Value	Per Ident (%)	Accession
Adhesion							
Sortase A, LPXTG specific	*SrtA*	678 703	2,297,851 2,161,679	100 100	0.0 0.0	100 99.86	CP011536.1 CP110798.1
Fibronectin-binding protein	*PrtF*	1693	2,100,449	100	0.0	99.79	CP002033.1
Peptide methionine sulfoxide reductase MsrA	*MsrA*	522 540	1,955,042 1,905,587	100 100	0.0 0.0	100 100	CP116618.1 CP039750.1
Peptide methionine sulfoxide reductase MsrB	*MsrB*	426	2,100,449	100	0.0	100	CP002033.1
Chaperonin (heat shock protein 33)	*Hsp33*	882	2,224,515	100	0.0	100	CP188318.1
Translation elongation factor Tu	*EF-Tu*	1191	2,068,538	100	0.0	99.83	CP124737.1
Exopolysaccharide							
Manganese-dependent protein-tyrosine phosphatase	*EpsB*	771	2,042,277	100	0.0	98.31	CP021964.1
Tyrosine-protein kinase EpsD	*EpsD*	741	2,089,202	100	0.0	96.22	LT906621.1
T yrosine-protein kinase transmembrane modulator EpsC	*EpsC*	771	2,114,971	100	0.0	99.09	CP121468.1
Undecaprenyl-phosphate galactosephosphotransferase	*EpsE*	666 654	2,089,202 1,905,587	100 100	0.0 0.0	97.45 99.69	LT906621.1 CP039750.1
Stress Resistance							
Cold shock							
Cold shock protein CspB	*CspB*	213	1,955,042	100	3e-105	100	CP116618.1
Cold shock protein CspA	*CspA*	201	2,161,679	100	1e-98	100	CP110798.1
PTS system, sucrose-specific IIA component	*EIIA*	1959	1,905,587	100	0.0	100	CP039750.1
Heat shock							
Ribosomal RNA small subunit methyltransferase E	*RsmE*	750	2,221,475	100	0.0	99.20	CP116755.1
DNA replication intiation control protein YabA	*YabA*	336	2,215,744	100	2e-173	100	CP118092.1
Heat-inducible transcription repressor HrcA	*HrcA*	1047	1,285,454	100	0.0	100	CP033371.1
Ribosomal protein L11 methyltransferase	*L11 + m*	963	2,173,659	100	0.0	99.79	CP184765.1
Figure 009886009886: phosphoesterase	*Figure 009886009886*	513	2,161,679	100	0.0	100	CP110798.1
tmRNA-binding protein SmpB	*SmpB*	474	2,100,449	100	0.0	99.79	CP002033.1
Translation elongation factor LepA	*Lep35A*	1833	2,315,729	100	0.0	99.95	CP170189.1
rRNA small subunit methyltransferase I	*RsmI*	861	2,297,851	100	0.0	99.88	CP011536.1
Nucleoside 5-triphosphatase RdgB (dHAPTP, dITP, XTP-specific) (EC 3.6.1.15)	*RdgB*	590 594	2,221,475 1,955,042	100 100	0.0 0.0	100 100	CP116755.1 CP116618.1
Chaperone protein DnaJ	*DnaJ-fam*	1161	2,221,475	100	0.0	100	CP116755.1
Chaperone protein DnaK	*DnaK*	1857	1,285,454	100	0.0	99.95	CP033371.1
Heat shock protein GrpE	*GrpE*	588	1,285,454	100	0.0	100	CP033371.1
GTP-binding protein HflX	*HflX*	1278	2,068,538	100	0.0	99.84	CP124737.1
Heat-inducible transcription repressor HrcA	*HrcA*	1047	1,285,454	100	0.0	100	CP033371.1
Heat shock protein 60 family co-chaperone GroES	*GroES*	282	1,955,042	100	8e-142	99.65	CP116618.1
Heat shock protein 60 family chaperone GroEL	*GroEL*	1632	2,101,878	100	0.0	99.69	CP050919.1
ATP-dependent Clp protease proteolytic subunit (EC 3.4.21.92)	*ClpP*	591	2,077,177	100	0.0	100	CP172330.1
ATP-dependent Clp protease, ATP-binding subunit ClpC	*ClpC*	2505	2,063,606	100	0.0	99.88	CP021790.1
ATP-dependent Clp protease ATP-binding subunit ClpX	*ClpX*	1251	2,042,277	100	0.0	99.92	CP021964.1
ClpB protein	*ClpB*	1881	2,297,851	100	0.0	100	CP011536.1
DedA protein	*DedA*	651 657	1,905,587 2,161,679	100 100	0.0 0.0	99.69 100	CP039750.1 CP110798.1
Osmotic stress							
Glycine betaine ABC transport system, permease protein OpuAB	*OpuAB*	645	1,955,042	100	0.0	100	CP116618.1
Choline binding protein A	*ChA*	1122	2,093,653	100	0.0	99.91	CP120668.1
L-proline glycine betaine binding ABC transporter protein ProX	*ProX*	903	2,224,515	100	0.0	100	CP188318.1
Glycine betaine ABC transport system, glycine betaine-binding protein OpuAC	*OpuAC*	645	1,955,042	100	0.0	100	CP116618.1
Oxidative stress							
Nicotinamidase	*PNC1*	576	2,221,475	100	0.0	100	CP116755.1
NAD-dependent protein deacetylase of SIR2 family	*SIR2*	1752	1,905,587	100	0.0	100	CP039750.1
Nicotinate phosphoribosyltransferase	*NPT1*	699	2,100,449	100	0.0	99.71	CP002033.1
NAD-dependent glyceraldehyde-3-phosphate dehydrogenase (EC 1.2.1.12)	*GAPDH_6*	1014	2,315,729	100	0.0	100	CP170189.1
Glutaredoxin-like protein NrdH, required for reduction of Ribonucleotide reductase class Ib	*NrdH*	222	2,101,878	100	3e-110	100	CP050919.1
Ferroxidase (EC 1.16.3.1)	*Fr*	549 468	1,905,587 2,100,449	100 100	0.0 0.0	99.82 100	CP039750.1 CP002033.1
Zinc uptake regulation protein ZUR	*ZUR*	450	1,955,042	100	0.0	99.78	CP116618.1
Peroxide stress regulator PerR, FUR family	*PerR2*	456	2,267,305	100	0.0	100	CP044354.1
Redox-sensitive transcriptional regulator (AT-rich DNA-binding protein)	*Rex*	651	1,955,042	100	0.0	100	CP116618.1
Iron-binding ferritin-like antioxidant protein	*IBP*	549 468	1,954,763 2,233,760	100 100	0.0 0.0	99.82 100	CP116617.1 CP102532.1
Non-specific DNA-binding protein Dps	*Dps*	1017	1,954,763	100	0.0	99.82	CP116617.1
Transcriptional regulator, Crp/Fnr family	*Crp*	645 636 651	2,094,354 2,298,221 2,149,913	100 100 100	0.0 0.0 0.0	100 100 100	CP065522.1 CP034099.1 CP082359.1
Peptide methionine sulfoxide reductase MsrA	*MsrA*	522 540	1,955,042 1,905,587	100 100	0.0 0.0	100 100	CP116618.1 CP039750.1
Bile salt tolerance							
Peptide methionine sulfoxide reductase MsrA	*MsrA*	522 540	1,955,042 1,905,587	100 100	0.0 0.0	100 100	CP116618.1 CP039750.1
HPr kinase/phosphorylase	*HPrK/P*	964	2,224,515	100	0.0	99.9	CP188318.1
Argininosuccinate synthase	*ArgA*	1291	1,973,978	100	0.0	99.85	AP024320.1
Acid tolerance							
dTDP-glucose 4,6-dehydratase		934 1043	2,086,671 2,032,186	100 100	0.0 0.0	98.61 99.32	CP101688.1 CP089305.1
dTDP-4-dehydrorhamnose reductase	*rmlD*	859	2,297,851	100	0.0	99.42	CP011536.1
Arginine deiminase	*ArcA*	1223	2,233,760	100	0.0	99.92	CP102532.1

#### Identification and Analysis of Bacteriocin Coding Sequences

**Fig. 5:**
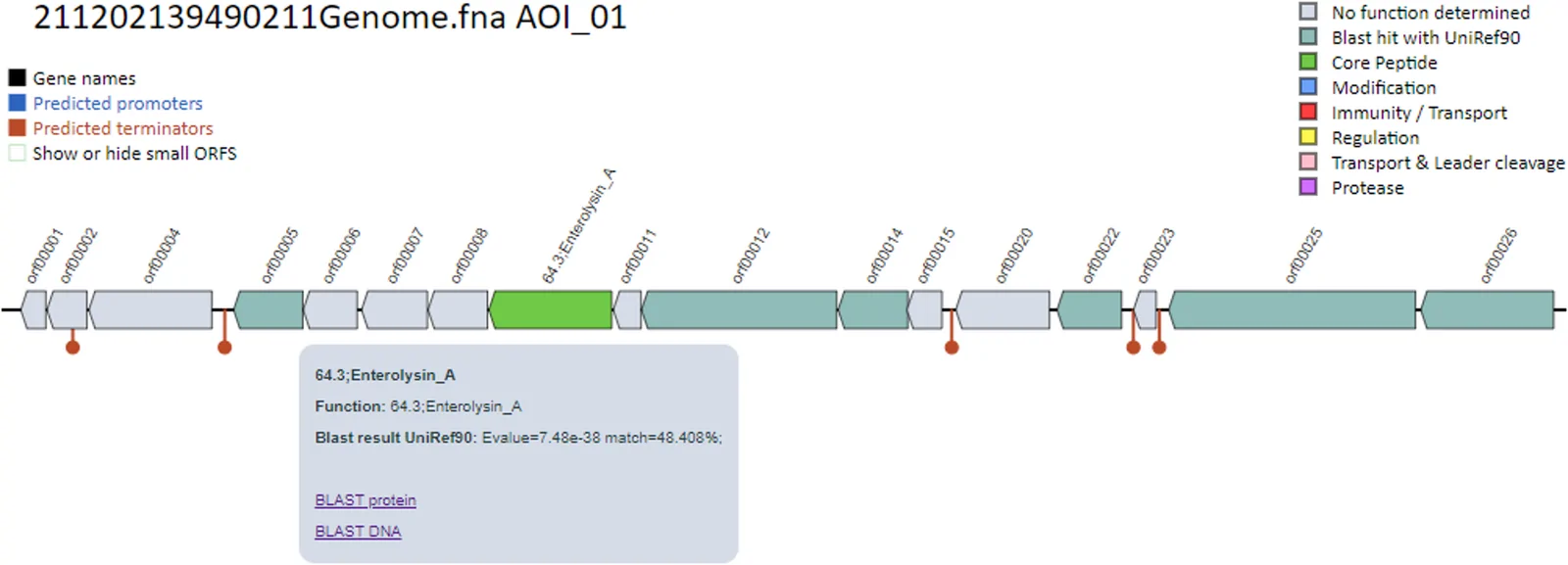
Prediction of bacteriocin structure in the genome of *L. fermentum* JAC 231 using the BAGEL5 program

Genes related to bacteriocin production were identified using BAGEL5. A sequence 48% similar to enterolysin A, a protein from *Enterococcus faecalis*, was identified. Figure 5 shows the position of the bacteriocin sequence in green.

#### Analysis of Genes Related to the Presence of Plasmids, Pathogenicity, Virulence and Resistance to Antimicrobials

Analysis of the *L. fermentum* JAC 231 genome using the RAST and PlasmidFinder tools did not reveal the presence of any mobile genetic elements. Using the PathogenFinder and VirulenceFinder algorithms, we did not identify pathogenicity genes or homologous virulence factors of common or clinically relevant pathogens, with the probability of being a human pathogen calculated as 0.203 using the PathogenFinder tool (Table S5).

The presence of resistance genes was assessed using RAST and ResFinder v. 4.0 and CARD. ResFinder v. 4.0 did not identify any resistance genes. The CARD program highlighted the presence of antibiotic resistance genes in the glycopeptide class (Table 3). The RAST tool revealed the presence of 20 coding sequences related to intrinsic antibiotic resistance, including two multidrug efflux pumps, one β-lactamase, four fluoroquinolone resistance determinants, and four ribosomal protection proteins associated with tetracycline resistance (Table 4).

**Table 3:** Resistance genes identified using the CARD tool for *L. fermentum* JAC 231 strain

CARD (v. RGI 5.1.0)					
RGI Criteria	**ARO** **Term**	**Detection criteria**	**AMR gene family**	**Drug Class**	**Resistance mechanism**
Strict	Gene vanT no cluster vanG	Protein Homologous Model	Glycopeptide resistance gene cluster, vanT Glycopeptide antibiotic	Glycopeptide antibiotic	Changing the antibiotic target

**Table 4:** Genes involved in resistance to antibiotics and toxic compounds

Gene Function RAST/BLAST	Gene (Abbrev.)	Query Length	Accession Length	Query Cover (%)	E Value	Per Ident (%)	Accession
Beta-lactamase							
Beta-lactamase class C and other penicillin binding proteins	*BLc*	1021 1017	2,298,221 2,149,913	100 100	0.0 0.0	99.9 100	CP034099.1 CP082359.1
Cobalt-zinc-cadmium resistance							
Cobalt-zinc-cadmium resistance protein	*CZCR*	552	2,001,184	100	0.0	100	CP091132.1
Cobalt-zinc-cadmium resistance protein CzcD	*CzcD*	907	2,042,277	100	0.0	99.56	CP021964.1
Probable cadmium-transporting ATPase (EC 3.6.3.3)	*PCT*	1931	2,221,475	100	0.0	99.84	CP116755.1
Transcriptional regulator, MerR family	*TRMer*	423 425 288 126	2,149,913 2,298,221 2,146,888 2,298,221	100 100 100 100	0.0 0.0 2e-141 1e-55	100 100 99.68 100	CP082359.1 CP034099.1 CP035054.1 CP034099.1
Copper homeostasis							
Copper chaperone	*CopZ*	231 229	2,077,616 2,068,538	100 100	3e-112 2e-108	100 99.56	CP119406.1 CP124737.1
Copper-translocating P-type ATPase (EC 3.6.3.4)	*CIA*	2286 2022	2,233,760 2,098,575	100 100	0.0 0.0	99.73 99,07	CP102532.1 *CP174186.1*
Multicopper oxidase	*MO*	1540	2,098,685	100	0.0	99.68	AP008937.1
Negative transcriptional regulator-copper transport operon	*TR*	459 195	2,267,305 81,588	100 100	0.0 3e-95	99.64 100	CP044354.1 CP096158.1
Mercuric reductase/Mercury resistance operon							
Mercuric ion reductase (EC 1.16.1.1)	*MerA*	1347	2,149,913	100	0.0	99.78	CP082359.1
PF00070 family, FAD-dependent NAD(P)-disulphide oxidoreductase	*PF00070*	1347	2,149,913	100	0.0	99.78	CP082359.1
Multidrug Resistance Efflux Pumps							
Multi antimicrobial extrusion protein (Na(+)/drug antiporter), MATE family of MDR efflux pumps	*MATE_all*	1320	2,001,184	100	0.0	99.92	CP091132.1
Multidrug-efflux transporter, major facilitator superfamily (MFS) (TC 2.A.1)	*MFS*	1693	2,238,401	100	0.0	99.84	CP045034.1
Resistance to fluoroquinolones							
DNA gyrase subunit A (EC 5.99.1.3)	*gyrA*	2511	2,034,355	100	0.0	100	CP095385.1
DNA gyrase subunit B (EC 5.99.1.3)	*gyrB*	1950	2,099,581	100	0.0	100	CP103293.1
Topoisomerase IV subunit A (EC 5.99.1.-)	*parC*	2478	2,267,305	100	0.0	99.96	CP044354.1
Topoisomerase IV subunit B (EC 5.99.1.-)	*parB*	1998	2,267,305	100	0.0	100	CP044354.1
Tetracycline resistance, ribosome protection type							
Ribosome protection-type tetracycline resistance related proteins, group 2	*Tet*-like2	1935	2,221,475	100	0.0	99.9	CP116755.1
Translation elongation factor G	*EF-G*	2085	2,165,664	100	0.0	100	CP160833.1

The presence of CRISPR-Cas sequences was investigated using the online program CRISPRCasFinder, which identified two hit sequences associated with the *cas* gene (Table S6).

The PHASTER server was used to predict the presence of prophage sequences in the *L. fermentum* JAC 231 genome. Based on oversight and potential prediction, this tool can identify intact, questionable, or incomplete prophage regions. A prophage region was identified throughout the genome, and analysis using the PHASTER tool revealed incomplete sequences.

### In Vitro Safety and Probiotic Activity

#### Antibiotic Susceptibility Assay

Antibiotic susceptibility profiles were assessed using disk diffusion method and the broth microdilution. *L. fermentum* JAC 231 was susceptible to most antibiotics tested. However, it showed resistance the vancomycin, and moderate sensitivity to ciprofloxacin (Table 5).

**Table 5:** Antimicrobial susceptibility testing results for *L. fermentum* JAC 231

Antimicrobials	*L. fermentum* JAC 231			
	Disk diffusion		Microdilution	
	Inhibition zone in mm (± SD) *	Interpretation*	MIC (µg/mL)	Activity**
Chloramphenicol	34.20 (± 0.84)	S		
Tetracycline	28.40 (± 1.14)	S		
Rifampicin	30.40 (± 3.44)	S		
Penicillin G	41.60 (± 1.14)	S		
Erythromycin	37.80 (± 1.64)	S		
Nitrofurantoin	39.60 (± 1.14)	S		
Gentamicin	14.20 (± 1.79)	S		
Cefazolin	41.20 (± 0.84)	S		
Clindamycin	30.40 (± 0.89)	S		
Sulfazotrim	16.40 (± 2.70)	S		
Oxacillin	13.75 (± 0.96)	S		
Novobiocin	40.40 (± 1.82)	S		
Teicoplanin	10.75 (± 1.50)	S		
Tigecycline	31.00 (± 1.58)	S		
Cefoxitin	27.80 (± 1.92)	S		
Linezolid	29.20 (± 1.92)	S		
Ciprofloxacin	14.00 (± 1.00)	MS		
Vancomycin	0	R	> 64	R

* Interpretation of results was based on Charteris et al. [49]. R: resistant; MS: moderately susceptible; S: sensitive. **Interpretative values for Vancomycin, according to the CLSI [50]: sensitive (S): ≤2 µg/mL; intermediate (I): 4–8 µg/mL; resistant (R): ≥16 µg/mL. All experiments were performed in triplicates in three independent experiments.

#### Tolerance to Gastrointestinal Conditions

Host survival under adverse gastrointestinal conditions was evaluated in vitro (Table 6). In the acid pH tolerance test (pH 2 and 4), the strains tested showed greater tolerance to pH 4 than to pH 2, where we observed a 31.15% reduction in the survival rate of the *L. fermentum* JAC 231 strain compared to the control (pH 7), and its growth was lower than that observed for *L. fermentum* ATCC 23,271 (*p* = 0.0005). We also evaluated survival at different concentrations of bile salts (0.5% and 1%) and compared it with that of control (without salts). *L. fermentum* JAC 231 showed a reduction in survival compared to the control without bile salts, but maintained stable viability at the tested concentrations of 5 g/L (87.34%) and 10 g/L (84.76%) (Table 6).

**Table 6:** Tolerance of *L. fermentum* JAC 231 and ATCC 23,271 to acidic pH and bile salts

Conditions	Survival % (± SD)^*^		*p*-value***
	*L. fermentum* JAC 231	*L. fermentum* ATCC 23271**	
pH 2.0	68.85 (± 0.81)	83.85 (± 2.23)	0.0077
pH 4.0	82.70 (± 0.71)	92.27 (± 1.37)	0.0005
Bile salts 5 g/L	87.34(± 0.76)	95.58 (± 0.92)	0.0003
Bile salts 10 g/L	84.76 (± 0.35)	75.37 (± 1.77)	0.0008

*The data represent the percentage survival of probiotic strains after 3 h of exposure to different gastrointestinal conditions compared to standard conditions. ** *L. fermentum* ATCC 23,271 was used as a positive control for the tests. ***Comparative analysis was performed using Student’s t test (*p* < 0.05).

#### Mucin and HeLa Cell Binding Assay

**Fig. 6:**
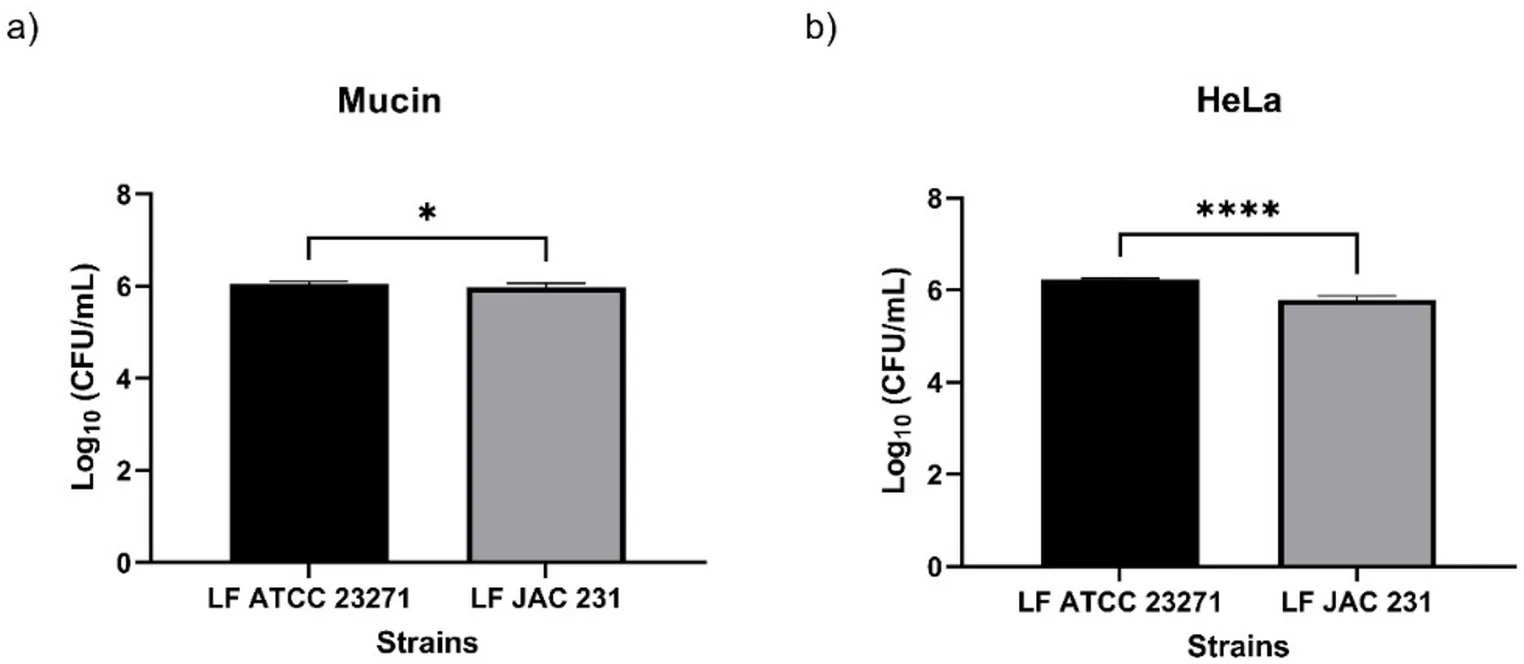
Quantification of the adhesion of *L. fermentum* JAC 231 (LF JAC 231) to HeLa cells (**a**) and mucin (**b**) relative to that of the control *L. fermentum* ATCC 23271 (LF ATCC 27271). The values are expressed as Log10 CFU/mL and represent the average of the obtained values. Legend: (*) *p*< 0.5 and (****) *p*<0.0001

The results of the binding assays for mucin and HeLa cells are presented as log_10_ CFU/mL (Fig. 6). In the mucin-binding assay (Fig. 6a), the *L. fermentum* ATCC 23,271 strain had a mean of 6.05 Log₁₀ CFU/mL, significantly higher than that of *L. fermentum* JAC 231 strain, which had 5.97 Log₁₀ CFU/mL (*p* < 0.05). In the HeLa cell adhesion test (Fig. 6b), we observed a similar result; the *L. fermentum* ATCC 23,271 strain showed 6.22 Log₁₀ CFU/mL, while the *L. fermentum* JAC 231 showed 5.79 Log₁₀ CFU/mL.

#### Antagonistic and Antimicrobial Activities of *L. fermentum* JAC 231 and its Cell-Free Supernatant

The inhibitory activity of *L. fermentum* JAC 231 against reference bacterial strains was determined using an antagonistic test with an overlay method. *L. fermentum* JAC 231 inhibited the growth of all bacteria tested, with zones of inhibition ranging from 16.60 ± 2.08 to 28.00 ± 2.88 mm (Table 7).

**Table 7:** Antimicrobial activity of *L. fermentum* JAC 231 and its cell-free supernatant

Strains	Inhibition zone in mm (± SD) *						
	Overlay method	CFS (untreated)	CFS pH-neutralized	CFS + proteinase K	CFS + Trypsin	MRS pH 4.0	MRS pH 6.0
*S. aureus* ATCC 25923	28.30 (± 2.88)	10.00 (± 1.00)	-	9.0 (± 1.00)	9.00 (± 1.00)	-	-
*E. faecalis* ATCC 29212	18.60 (± 1.15)	-	-	-	-	-	-
*K. pneumoniae* ATCC 700603	21.00 (± 3.21)	-	-	-	-	-	-
*P. aeruginosa* ATCC 27853	26.00 (± 1.73)	14.70 (± 0.57)	-	10.33 (± 0.57)	10.33 (± 1.15)	-	-
EPEC E2348/69	24.40 (± 2.88)	-	-	-	-	-	-
*E. coli* ATCC 25922	16.60 (± 2.08)	10.30 (± 0.57)	-	8.60 (± 0.57)	8.33 (± 0.57)	-	-
*S. enteriditis* ATCC 13076	26.60 (± 2.88)	-	-	-	-	-	-

*Values represent the mean in mm (± standard deviation - SD) of triplicate assays performed on three separate days. (–) indicates no detectable inhibition.

The antimicrobial activity of the cell-free supernatant (CFS) was evaluated using the agar diffusion assay. The untreated CFS exhibited inhibitory activity against S*taphylococcus aureus*, *Pseudomonas aeruginosa*, and *Escherichia coli*, with inhibition zones ranging from 10.00 ± 1.00 to 14.70 ± 0.57 mm (Table 7). Neutralization of CFS resulted in a complete loss of inhibitory activity, whereas treatment with proteinase K or trypsin did not abolish inhibition, indicating that the antimicrobial effect is primarily mediated by acidic compounds rather than proteinaceous substances.

#### Inhibition of Pathogen Adhesion to HeLa Cells

**Fig. 7:**
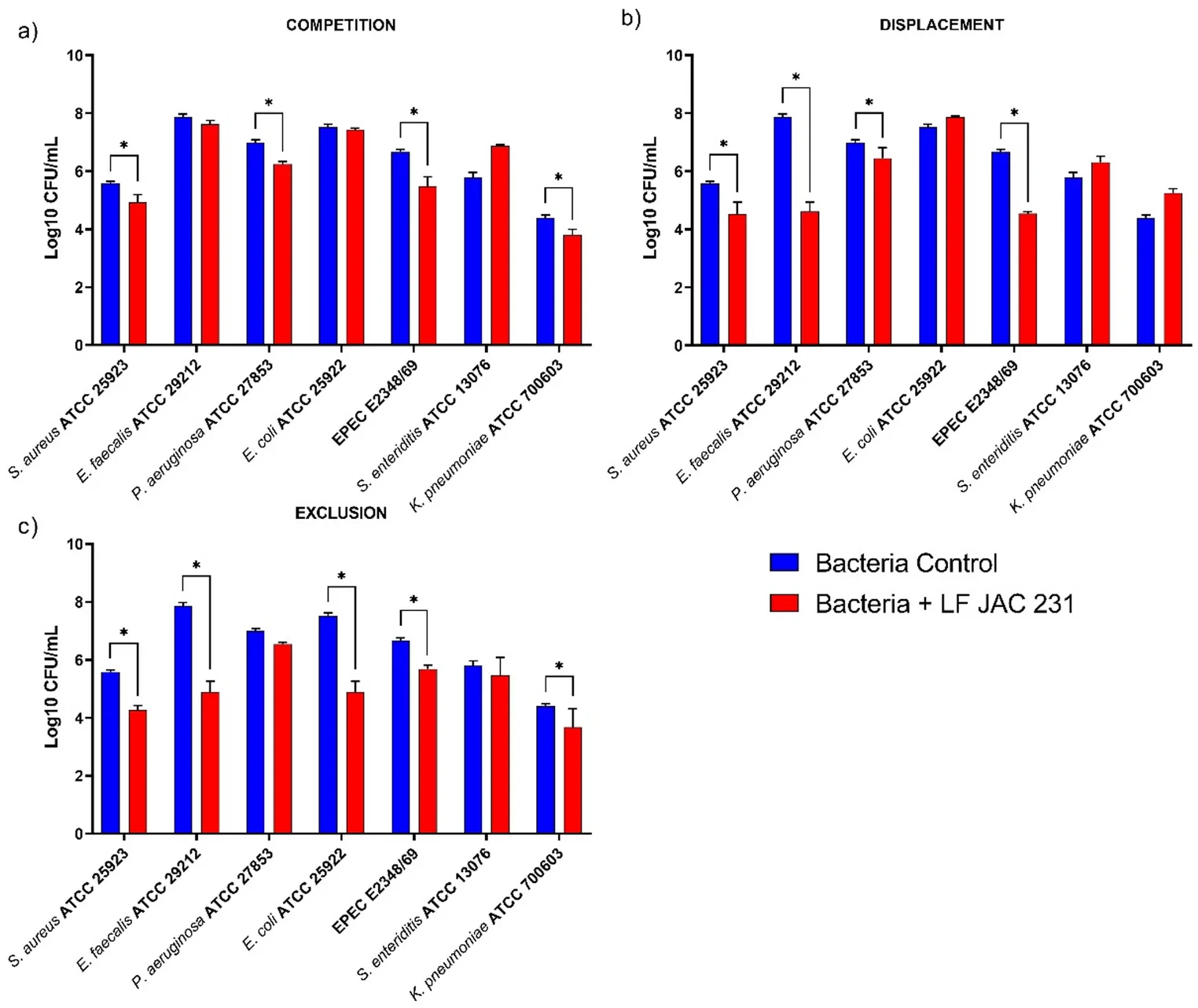
Inhibition of pathogen adhesion to HeLa cells by *L. fermentum* JAC 231 in three experiments: (**a**) competition, (**b**) displacement, and (**c**) exclusion. Two-way ANOVA followed by Dunnet analysis. * *p* < 0.05

The effect of *L. fermentum* JAC 231 on the adhesion of bacterial strains to HeLa cells was evaluated using three methods: competition, exclusion, and displacement (Fig. 7a-c). In the competition assays, the tested probiotic strains reduced the adhesion of *S. aureus* ATCC 25923, *P. aeruginosa* ATCC 27853, EPEC E2348/69, and *K. pneumoniae* ATCC 700603 (Fig. 7a). In the displacement assays, where the probiotic strain was introduced after the adhesion of pathogenic bacteria, we observed better interference with the adhesion of *S. aureus* ATCC 25923, *E. faecalis* ATCC 29212, *P. aeruginosa* ATCC 27853, and EPEC E2348/69 (Fig. 7b).

In the displacement experiments, we observed a reduction in four bacterial strains (*S. aureus* ATCC 25923, *E. faecalis* ATCC 29212, *P. aeruginosa* ATCC 27853, and EPEC E2348/69) (Fig. 7c). In all tests, *S. enteritidis* ATCC 13076 adhered more or had a CFU/mL count equivalent to that of the control, which contained only the bacteria, when incubated in the presence of *L. fermentum* JAC 231.

## Discussion

This study presents a comprehensive genomic and phenotypic evaluation of *L. fermentum* JAC 231, a strain previously isolated from the vaginal microbiota of an asymptomatic woman [28]. The findings build on earlier evidence of anti-*Candida* activity by demonstrating probiotic potential through genomic insights, stress resilience, adhesion capacity, antimicrobial effects, and safety. Together, these data contribute to the growing evidence base positioning *L. fermentum* strains as promising candidates for therapeutic and prophylactic use as probiotics in humans.

The genomic data of *L. fermentum* JAC 231 revealed a genome of approximately 2.06 Mbp with GC content of 51.4%. These features are consistent with those of previously sequenced *L. fermentum* strains, such as ATCC 23,271 and AGR1485 [1, 57–59]. Phylogenetic analysis based on core genome alignments confirmed that JAC 231 clustered closely with strains known for their probiotic and health-promoting effects, including AGR 1485, CECT 5716, MCC 2760, and IFO 3976. This evolutionary proximity supports the hypothesis that JAC 231 shares conserved probiotic determinants with these strains [37, 60].

Comparative genomics revealed that *Limosilactobacillus fermentum* JAC 231 shares 1,587 orthologous clusters with other probiotic strains of the species, including ATCC 23271 (reported anti-*Candida* activity) and CECT 5716 (known immunomodulatory properties) [10, 21, 61]. Only one lineage-specific cluster encoding two hypothetical proteins was identified, indicating that strain-specific genetic determinants are limited. Therefore, the particular features of JAC 231 are unlikely to be explained by unique genes but rather by its functional phenotype, potentially shaped by its ecological origin and the specific combination of conserved traits it expresses, reinforcing the need to integrate genomic insights with experimental validation.

In contrast to strains such as *L. fermentum* CECT 5716, originally isolated from human milk, and ATCC 23271, obtained from the gastrointestinal tract, JAC 231 was recovered from the vaginal microbiota of a healthy asymptomatic woman [28]. This ecological niche may contribute to adaptive traits associated with persistence and microbial interactions in the urogenital environment. Consistent with this possibility, JAC 231 exhibited a dual functional profile characterized by broad antibacterial activity and marked anti-*Candida* effects. In our previous study, JAC 231 significantly reduced the virulence of *C. albicans*, including hyphal formation, biofilm development, and adhesion to epithelial cells [28].

The present findings extend this functional profile by demonstrating its activity against several bacterial pathogens. In competition, exclusion, and displacement assays, JAC 231 reduced the adhesion of both Gram-positive and Gram-negative bacteria, including *Staphylococcus aureus*, *Enterococcus faecalis*, *Pseudomonas aeruginosa*, and *Escherichia coli*. This interference likely reflects multiple mechanisms, including organic acid production, the presence of genes potentially associated with bacteriocin-related activity (e.g., a sequence with 48% identity to enterolysin A), and anti-adhesive interactions with epithelial surfaces. Taken together, the vaginal origin of JAC 231 and its combination of antagonistic mechanisms against both fungal and bacterial pathogens highlight a distinct functional profile that may differentiate this strain from other *L. fermentum* isolates and support its potential as a niche-adapted probiotic for urogenital health.

In this study, we used data mining of the genomic sequences of *L. fermentum* JAC 231 to discover genes that encode proteins responsible for adhesion, survival, and adaptation to adverse gastrointestinal environments, traits that are associated with probiotic functions. These include genes involved in the biosynthesis of essential B-complex vitamins (thiamine, riboflavin, pyridoxine, biotin, and folate), which not only support host metabolism but also enhance microbial competitiveness in the gastrointestinal tract [62–64]. The presence of multiple genes related to oxidative stress tolerance, heat shock proteins, osmotic stress resistance, and acid and bile tolerance further suggests that *L. fermentum* JAC 231 can endure harsh conditions encountered during gastrointestinal transit, which is a prerequisite for probiotic efficacy [22, 65].

Importantly, the genome encodes key adhesion-related proteins, such as Sortase A, fibronectin-binding proteins, and LPxTG motif-containing surface proteins. These molecules play essential roles in the adhesion of probiotic bacteria to epithelial surfaces and mucins, facilitating colonization and immunomodulatory interactions in the gut [66, 67]. Although *L. fermentum* JAC 231 exhibited lower in vitro adhesion to mucin and HeLa cells than the reference strain *L. fermentum* ATCC 23,271, this finding does not necessarily contradict its probiotic potential. Adhesion is strain-specific and multifactorial, and *L. fermentum* JAC 231 may exert anti-adhesive effects via competitive exclusion, steric hindrance or modulation of host cell receptors [10].

Indeed, despite modest adhesion, *L. fermentum* JAC 231 significantly inhibited the adhesion of pathogens such as *S. aureus*, *E. faecalis*, *P. aeruginosa*, and EPEC to epithelial cells. This effect was observed in competitive, exclusion, and displacement assays, indicating a robust ability to interfere with pathogen colonization of the host. Such anti-adhesive activity is supported by the presence of adhesin-encoding genes and the production of exopolysaccharides, which may form steric barriers or promote host cell signaling that suppresses pathogen attachment [66–68].

JAC 231 also displayed strong antimicrobial activity in agar diffusion assays against a range of pathogens, including Gram-positive and Gram-negative bacteria. Experimental evaluation using treated CFS demonstrated that this inhibitory effect was predominantly mediated by organic acid production, as antimicrobial activity was abolished after pH neutralization and was not affected by protease treatment. These results indicate that the acidic compounds are responsible for the antimicrobial activity observed under the tested conditions [69]. In the BAGEL 5 database, a cluster of genes encoding bacteriocin enterolysin A was detected with 48% sequence identity. Enterolysin A is a class III bacteriocin initially identified in *Enterococcus faecalis* and other LAB, such as *Lactobacillus plantarum* and *Lactococcus lactis* [70, 71]. Initial analyses revealed that the antimicrobial activity of this bacteriocin against gram-positive bacteria affects cell wall degradation [70, 72]. The presence of this bacteriocin in other *L. fermentum* strains has also been reported. Santos et al. [59] demonstrated that the *L. fermentum* ATCC 23,271 strain presented a coding region for a protein similar to enterolysin A, which, through three algorithms of the CAMPR3 tool, indicated a high probability of possessing antimicrobial activity.

In the evaluation of probiotic candidates, it is crucial to assess potential risks, particularly the possibility of antibiotic resistance mechanisms and horizontal gene transfer to other microorganisms [20, 73]. To address these concerns, in vitro testing and, more recently, genome analysis have been employed to assess the safety and other factors related to pathogenicity and virulence [60, 74]. Analysis of the *L. fermentum* JAC 231 genome using RAST, PlasmidFinder, PathogenFinder, and VirulenceFinder tools did not reveal the presence of mobile genetic elements or pathogenicity-related genes. However, CARD and RAST tools indicated the presence of glycopeptide-class antibiotic resistance genes (vancomycin).

Vancomycin resistance is one of the most well-documented antibiotic resistance mechanisms within the LAB group and is considered intrinsic and non-transferable [73, 75]. In our antibiotic susceptibility testing, *L. fermentum* JAC 231 demonstrated moderate sensitivity to ciprofloxacin and resistance to vancomycin, as previously reported using in silico tools. This finding is consistent with that of Anisimova and Yarullina [76], who studied the antibiotic resistance patterns of 20 strains of *Lactobacillus*, including *L. fermentum*, and found that the majority of the isolates were resistant to vancomycin, ciprofloxacin, and aminoglycosides, with five strains showing resistance to these antibiotics. These results indicate a strong relationship between genotype and phenotype, as previously reported [59, 75, 76], highlighting the need for tools that combine genotypic and phenotypic analyses to evaluate the safety of probiotic candidates [24, 59].

*L. fermentum* JAC 231 exhibited an incomplete prophage region, which is likely non-functional, as well as two CRISPR sequences connected to the Cas genes. CRISPR‒Cas genes serve as an essential defense mechanism for bacterial cells, preventing the spread of infections by bacteriophages and the insertion of foreign DNA into the bacterial genome [77]. Together, these findings strongly support the safety profile and suitability of this strain for probiotic use, especially considering the increasing concern over antimicrobial resistance dissemination.

From a broader perspective, the probiotic profile of JAC 231 reflects the characteristics of other well-studied *L. fermentum* strains. For instance, CECT 5716, a strain used in commercial formulations, has shown efficacy in preventing infections and modulating immune response [64, 78]. MCC 2760, another clinically evaluated strain, exhibits anti-inflammatory, antidiabetic, and cholesterol-lowering effects [79–82]. The genomic and phenotypic similarities between JAC 231 and these strains strengthen the rationale for its development as a multifunctional probiotic.

However, this study had some important limitations. First, all probiotic traits were assessed using in vitro assays and bioinformatic analysis. Although informative, these approaches do not capture the complexity of host-microbe interactions, immune responses, or ecological competition within the human body [83, 84]. Second, gene expression under host-relevant conditions remains unexplored. It is essential to validate whether the identified genes are actively transcribed and translated in vivo in humans. Third, antimicrobial assays, while indicative, do not confirm therapeutic efficacy in situ, where complex variables such as pH, enzymatic degradation, and microbiota interactions play key roles.

In addition, the long-term colonization potential and technological stability of this strain have not been evaluated. These aspects are crucial for the design of probiotic products and their regulatory approval. Future investigations should incorporate animal models, human clinical trials, and functional studies on gene expression, bioactive compound production and host immune modulation.

## Conclusion

*L. fermentum* JAC 231 displays a compelling combination of genomic, phenotypic, and functional features that support its potential as a probiotic. It possesses a stable genome enriched with genes related to stress tolerance, vitamin biosynthesis, adhesion, and immune system interaction. Phenotypically, the strain survived acidic and bile stress, inhibited the adhesion of major pathogens, and exhibited broad-spectrum antimicrobial activity. Its in silico and in vitro safety profiles further underscore its suitability for therapeutic applications.

The phylogenetic proximity of JAC 231 to other validated probiotic strains, coupled with its multifunctional traits, positions it as a strong candidate for clinical development for the treatment of mucosal infections, biofilm-associated diseases, and possibly metabolic or inflammatory disorders. However, robust in vivo validation, mechanistic studies, and technological assessments are required before clinical application. Addressing these gaps is pivotal in determining the true potential of this strain as a safe, effective, versatile probiotic agent.

## Supplementary Information

Below is the link to the electronic supplementary material.

Supplementary Material 1 (PDF 1.25 MB)

## Data Availability

The genomic data of *L. fermentum* JAC 231 was submitted to NCBI (National Centre for Biotechnology Information) under the accession number JAVFHY000000000. Raw sequencing reads (FASTQ files) are available in the NCBI Sequence Read Archive (SRA) under accession number SRR37146884. The BioProject accession number PRJNA1007394 and BioSample is SAMN37068468.
