# Supplementary material for: Comprehensive Genomic Characterization and Probiotic Properties Evaluation of Limosilactobacillus fermentum JAC 231 Isolated from Vaginal Microbiota
Source: Probiotics Antimicrob Proteins. 2026 Apr 28;18(7):8512–31. doi: 10.1007/s12602-026-11025-7 (PMC13518392; doi:10.1007/s12602-026-11025-7)
Supplement: Supplementary file 1 [file 12602_2026_11025_MOESM1_ESM.pdf]

Journal: Probiotics and Antimicrobial Proteins

Comprehensive Genomic Characterization and Probiotic Properties Evaluation of *Limosilactobacillus fermentum* JAC 231 Isolated from Vaginal Microbiota

Carmem Duarte Lima Campos[1], Amanda Graziella Goncalves Mendes[1], José Lima Pereira-Filho[1], Viviane da Silva Sousa Almeida[1], Ana Beatriz Santos Sousa[1], Israel Viegas Moreira[1], João Lucas do Carmo Lima[1], Aleania Polassa Almeida Pereira[1], Allysson Kayron Carvalho Silva[2], Laís Araújo Souza Wolff[2], Kátia Regina Assunção Borges[2], Cristianne Roberta Rhoden[3], Cinara Regina Aragão Vieira Monteiro[1], Cristina de Andrade Monteiro[4], Elizabeth Soares Fernandes[5], Valério Monteiro-Neto[1*].

[1] Programa de Pós-Graduação em Ciências da Saúde – PPGCS, Centro de Ciências Biológicas e da Saúde, Universidade Federal do Maranhão. Avenida dos Portugueses, 1966, campus Bacanga, São Luís, MA, 65080-040, Brazil;

[2] Programa de Doutorado em Biotecnologia – Rede Nordeste de Biotecnologia (RENORBIO). Universidade Federal do Maranhão. Avenida dos Portugueses, 1966, campus Bacanga, São Luís, MA, 65080-040, Brazil;

[3] Laboratório Cedro, Avenida Silva Maia, 81, Centro, São Luís MA, 65020-570, Brazil

[4] Instituto Federal de Educação Ciência e Tecnologia do Maranhão, Avenida Getúlio Vargas, 4, Monte Castelo, São Luís, 65030-005, MA, Brazil.

[5] Department of Animal and Veterinary Sciences - ANIVET Ruminant Nutrition (RUN), Aarhus University, Denmark

* Correspondence to: valerio.monteiro@ufma.br.

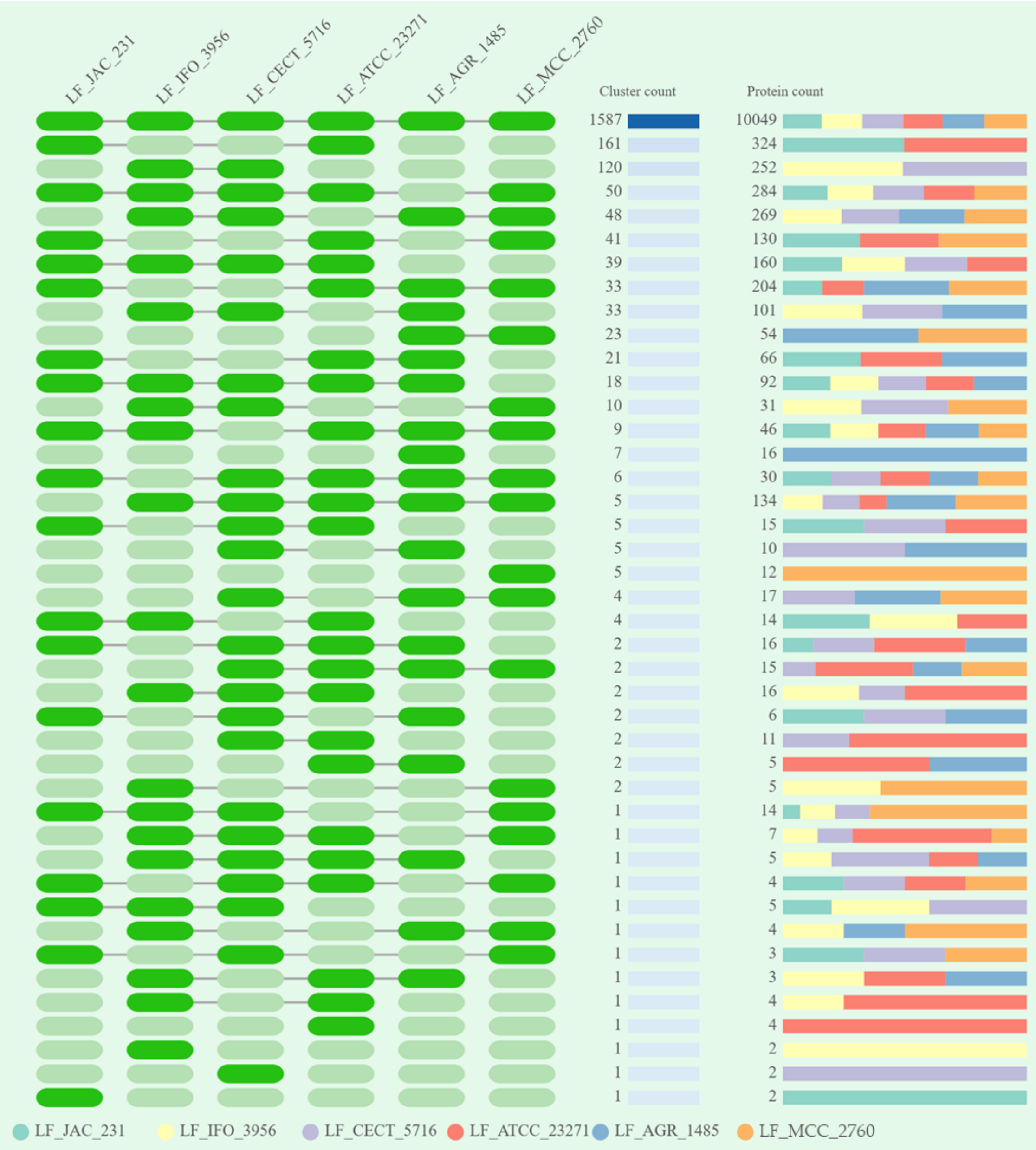

Figure S1. Orthologous cluster analysis of the six strains of *L. fermentum*: JAC 231, AGR 1485, ATCC 23271, CECT 5716, IFO 3956, and MCC 2760.

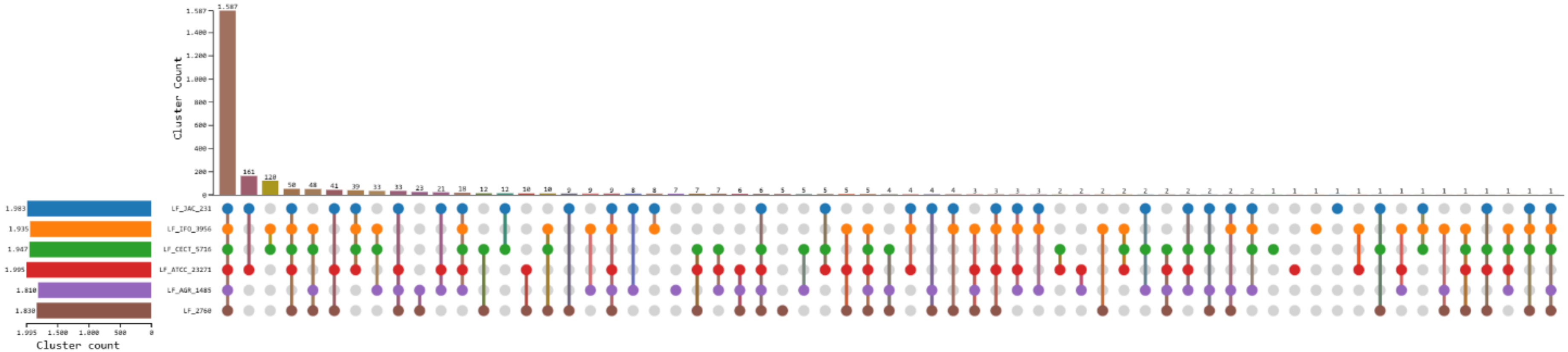

Figure S2. Distribution of orthogroups among *L. fermentum* strains. The plot highlights the core genome, which is composed of 1,587 conserved orthogroups, as well as species-specific adaptations. The strain *L. fermentum* JAC 231 contains 1,983 orthogroups, including one unique cluster. The presence of unique and subset-specific orthogroups suggests functional specialization, reflecting the evolutionary divergence among strains.

Table S1. RAST Subsystems and features of *L. fermentum* JAC 231

Category - subcategory - Subsystem	Gene count
**Amino Acids and Derivatives**	
**Alanine, serine, and glycine**	
Alanine biosynthesis	3
Glycine and Serine Utilization	8
Glycine Biosynthesis	1
Serine Biosynthesis	3
**Amino Acids and Derivatives - no subcategory**	
Creatine and Creatinine Degradation	1
**Arginine; urea cycle, polyamines**	
Arginine and Ornithine Degradation	7
Arginine Biosynthesis -- gjo	10
Arginine Biosynthesis extended	10
Arginine Deiminase Pathway	5
**Aromatic amino acids and derivatives**	
Common Pathway For Synthesis of Aromatic Compounds (DAHP synthase to chorismate)	2
Phenylalanine and Tyrosine Branches from Chorismate	3
**Glutamine, glutamate, aspartate, asparagine; ammonia assimilation**	
Glutamate and Aspartate uptake in Bacteria	2
Glutamine synthetases	1
Glutamine, Glutamate, Aspartate and Asparagine Biosynthesis	7
**Lysine, threonine, methionine, and cysteine**	
Lysine Biosynthesis DAP Pathway	9
Lysine Biosynthesis DAP Pathway, GJO scratch	9
Methionine Biosynthesis	11
Methionine Degradation	7
S-methylmethionine	2
Threonine and Homoserine Biosynthesis	6
Threonine degradation	1
**Proline and 4-hydroxyproline**	
Proline Synthesis	3
**Carbohydrates**	
**Central carbohydrate metabolism**	
Dehydrogenase complexes	3
Glycolate, glyoxylate interconversions	3
Glycolysis and Gluconeogenesis	8
Pentose phosphate pathway	8
Pyruvate Alanine Serine Interconversions	4
Pyruvate metabolism I: anaplerotic reactions, PEP	5
Pyruvate metabolism II: acetyl-CoA, acetogenesis from pyruvate	8
**Di- and oligosaccharides**	
Lactose and Galactose Uptake and Utilization	9
Sucrose utilization	5

Trehalose Biosynthesis	1
Trehalose Uptake and Utilization	2
**Fermentation**	
Acetoin, butanediol metabolism	2
Acetyl-CoA fermentation to Butyrate	4
Fermentations: Lactate	5
**Monosaccharides**	
Deoxyribose and Deoxynucleoside Catabolism	3
D-galactarate, D-glucarate and D-glycerate catabolism	3
D-galactarate, D-glucarate and D-glycerate catabolism - gjo	3
D-gluconate and ketogluconates metabolism	4
D-ribose utilization	4
Mannose Metabolism	3
Xylose utilization	5
**One-carbon Metabolism**	
One-carbon metabolism by tetrahydropterines	4
**Organic acids**	
Alpha-acetolactate operon	2
Glycerate metabolism	5
Lactate utilization	4
**Sugar alcohols**	
Glycerol and Glycerol-3-phosphate Uptake and Utilization	5
**Cell Division and Cell Cycle**	
**Cell Division and Cell Cycle - no subcategory**	
Bacterial Cytoskeleton	16
Control of cell elongation - division cycle in Bacilli	7
Macromolecular synthesis operon	7
YgjD and YeaZ	2
**Checkpoint control**	
Bacterial checkpoint-control-related cluster	4
**Cell Wall and Capsule**	
**Capsular and extracellular polysacchrides**	
dTDP-rhamnose synthesis	4
Exopolysaccharide Biosynthesis	4
Rhamnose containing glycans	5
Sialic Acid Metabolism	5
**Cell Wall and Capsule - no subcategory**	
Murein Hydrolases	3
Peptidoglycan Biosynthesis	16
Peptidoglycan biosynthesis--gjo	6
Recycling of Peptidoglycan Amino Acids	1
UDP-N-acetylmuramate from Fructose-6-phosphate Biosynthesis	6
YjeE	4
**Gram-Positive cell wall components**	
D-Alanyl Lipoteichoic Acid Biosynthesis	4
Polyglycerolphosphate lipoteichoic acid biosynthesis	5
Sortase	1

Teichoic and lipoteichoic acids biosynthesis	7
Teichuronic acid biosynthesis	1
**Cofactors, Vitamins, Prosthetic Groups, Pigments**	
**Biotin**	
Biotin biosynthesis	3
**Coenzyme A**	
Coenzyme A Biosynthesis	7
**Cofactors, Vitamins, Prosthetic Groups, Pigments - no subcategory**	
Thiamin biosynthesis	8
**Folate and pterines**	
5-FCL-like protein	12
Folate Biosynthesis	10
Folate biosynthesis cluster	7
Molybdenum cofactor biosynthesis	11
**Lipoic acid**	
Lipoic acid metabolism	1
**NAD and NADP**	
NAD and NADP cofactor biosynthesis global	8
**Pyridoxine**	
Pyridoxin (Vitamin B6) Biosynthesis	6
Pyridoxin(Vitamin B6) Degradation Pathway	1
**Riboflavin, FMN, FAD**	
Flavodoxin	3
riboflavin to FAD	4
Riboflavin, FMN and FAD metabolism	9
Riboflavin, FMN and FAD metabolism in plants	12
**Tetrapyrroles**	
Heme and Siroheme Biosynthesis	3
**DNA Metabolism**	
**CRISPs**	
CRISPRs	7
**DNA Metabolism - no subcategory**	
DNA structural proteins, bacterial	2
Restriction-Modification System	3
Type I Restriction-Modification	3
YcfH	1
**DNA recombination**	
RuvABC plus a hypothetical	3
**DNA repair**	
ATP-dependent Nuclease	2
DNA Repair Base Excision	7
DNA repair system including RecA, MutS and a hypothetical protein	2
DNA repair, bacterial	14
DNA repair, bacterial DinG and relatives	1
DNA repair, bacterial MutL-MutS system	3
DNA repair, bacterial RecBCD pathway	1
DNA repair, bacterial RecFOR pathway	8

DNA repair, bacterial UvrD and related helicases	1
DNA repair, UvrABC system	4
Uracil-DNA glycosylase	2
**DNA replication**	
DNA topoisomerases, Type I, ATP-independent	2
DNA topoisomerases, Type II, ATP-dependent	4
**DNA uptake, competence**	
DNA processing cluster	5
Gram Positive Competence	7
**Dormancy and Sporulation**	
**Dormancy and Sporulation - no subcategory**	
Sporulation-associated proteins with broader functions	1
**Fatty Acids, Lipids, and Isoprenoids**	
**Fatty acids**	
Fatty Acid Biosynthesis FASII	11
Fatty acid metabolism cluster	2
**Fatty Acids, Lipids, and Isoprenoids - no subcategory**	
Polyhydroxybutyrate metabolism	4
**Isoprenoids**	
Isoprenoid Biosynthesis	10
Isoprenoinds for Quinones	2
Mevalonate Branch of Isoprenoid Biosynthesis	6
**Phospholipids**	
Cardiolipin synthesis	1
Glycerolipid and Glycerophospholipid Metabolism in Bacteria	13
**Triacylglycerols**	
Triacylglycerol metabolism	1
**Iron acquisition and metabolism**	
**Iron acquisition and metabolism - no subcategory**	
Encapsulating protein for DyP-type peroxidase and ferritin-like protein oligomers	1
**Membrane Transport**	
**Cation transporters**	
Copper Transport System	1
Magnesium transport	2
Transport of Nickel and Cobalt	1
**Protein translocation across cytoplasmic membrane**	
Bacterial signal recognition particle (SRP)	3
EcsAB transporter affecting expression and secretion of secretory preproteins	4
**Uni- Sym- and Antiporters**	
Proton-dependent Peptide Transporters	1
**Metabolism of Aromatic Compounds**	
**Peripheral pathways for catabolism of aromatic compounds**	
Biphenyl Degradation	2
**Miscellaneous**	
**Miscellaneous - no subcategory**	
Broadly distributed proteins not in subsystems	2

DedA family of inner membrane proteins	1
Muconate lactonizing enzyme family	1
Phosphoglycerate mutase protein family	4
**Plant-Prokaryote DOE project**	
Conserved gene cluster possibly involved in RNA metabolism	2
Iron-sulfur cluster assembly	7
**Nitrogen Metabolism**	
**Denitrification**	
Denitrifying reductase gene clusters	4
**Nitrogen Metabolism - no subcategory**	
Nitrate and nitrite ammonification	5
Nitrosative stress	1
**Nucleosides and Nucleotides**	
**Detoxification**	
Housecleaning nucleoside triphosphate pyrophosphatases	4
Nudix proteins (nucleoside triphosphate hydrolases)	2
**Nucleosides and Nucleotides - no subcategory**	
Ribonucleotide reduction	7
**Purines**	
De Novo Purine Biosynthesis	15
Purine conversions	15
Purine Utilization	3
Xanthine Metabolism in Bacteria	2
**Pyrimidines**	
De Novo Pyrimidine Synthesis	9
pyrimidine conversions	11
**Phages, Prophages, Transposable elements, Plasmids**	
**Phages, Prophages**	
Phage capsid proteins	2
Phage packaging machinery	3
Phage replication	3
**Phosphorus Metabolism**	
**Phosphorus Metabolism - no subcategory**	
High affinity phosphate transporter and control of PHO regulon	7
Phosphate metabolism	10
Polyphosphate	2
**Potassium metabolism**	
**Potassium metabolism - no subcategory**	
Potassium homeostasis	5
**Protein Metabolism**	
**Protein biosynthesis**	
Glycyl-tRNA synthetase	2
Programmed frameshift	1
Ribosome activity modulation	1
Ribosome biogenesis bacterial	11
Translation elongation factor G family	2
Translation elongation factors bacterial	5

Translation initiation factors bacterial	5
Translation termination factors bacterial	9
tRNA aminoacylation, Ala	1
tRNA aminoacylation, Arg	1
tRNA aminoacylation, Asp and Asn	5
tRNA aminoacylation, Cys	1
tRNA aminoacylation, Glu and Gln	5
tRNA aminoacylation, Gly	2
tRNA aminoacylation, His	1
tRNA aminoacylation, Ile	1
tRNA aminoacylation, Leu	1
tRNA aminoacylation, Lys	1
tRNA aminoacylation, Met	1
tRNA aminoacylation, Phe	3
tRNA aminoacylation, Pro	1
tRNA aminoacylation, Ser	1
tRNA aminoacylation, Thr	1
tRNA aminoacylation, Trp	1
tRNA aminoacylation, Tyr	1
tRNA aminoacylation, Val	1
tRNAs	12
Universal GTPases	17
**Protein degradation**	
Metallocarboxypeptidases (EC 3.4.17.-)	1
Protein degradation	2
Proteolysis in bacteria, ATP-dependent	7
**Protein folding**	
GroEL GroES	6
Peptidyl-prolyl cis-trans isomerase	1
Protein chaperones	4
**Protein processing and modification**	
Lipoprotein Biosynthesis	2
Peptide methionine sulfoxide reductase	2
Signal peptidase	2
**Regulation and Cell signaling**	
**Regulation and Cell signaling - no subcategory**	
cAMP signaling in bacteria	3
Cell envelope-associated LytR-CpsA-Psr transcriptional attenuators	1
HPr catabolite repression system	2
LysR-family proteins in Escherichia coli	1
Sex pheromones in Enterococcus faecalis and other Firmicutes	3
Stringent Response, (p)ppGpp metabolism	1
**Respiration**	
**Electron accepting reactions**	
Anaerobic respiratory reductases	3
Terminal cytochrome d ubiquinol oxidases	4
Terminal cytochrome oxidases	4

**Electron donating reactions**	
Respiratory dehydrogenases 1	3
Succinate dehydrogenase	1
**RNA Metabolism**	
**RNA processing and modification**	
16S rRNA modification within P site of ribosome	6
Ribonuclease H	3
Ribonucleases in Bacillus	5
RNA methylation	12
RNA processing and degradation, bacterial	4
RNA pseudouridine syntheses	6
tRNA modification Bacteria	19
tRNA nucleotidyltransferase	1
tRNA processing	7
**Transcription**	
RNA polymerase bacterial	5
Rrf2 family transcriptional regulators	1
Transcription factors bacterial	9
Transcription initiation, bacterial sigma factors	1
**Stress Response**	
**Cold shock**	
Cold shock, CspA family of proteins	2
**Detoxification**	
Uptake of selenate and selenite	1
**Heat shock**	
Heat shock dnaK gene cluster extended	12
**Osmotic stress**	
Choline and Betaine Uptake and Betaine Biosynthesis	4
**Oxidative stress**	
Glutaredoxins	1
Glutathione: Biosynthesis and gamma-glutamyl cycle	1
Glutathione: Redox cycle	1
Oxidative stress	7
Redox-dependent regulation of nucleus processes	4
**Stress Response - no subcategory**	
Dimethylarginine metabolism	1
Hfl operon	1
**Sulfur Metabolism**	
**Sulfur Metabolism - no subcategory**	
Galactosylceramide and Sulfatide metabolism	3
Thioredoxin-disulfide reductase	4
**Virulence, Disease and Defense**	
**Adhesion**	
Streptococcus pyogenes recombinatorial zone	2
**Invasion and intracellular resistance**	
Mycobacterium virulence operon involved in an unknown function with a Jag Protein and YidC and YidD	2

Mycobacterium virulence operon involved in DNA transcription	2
Mycobacterium virulence operon involved in protein synthesis (LSU ribosomal proteins)	3
Mycobacterium virulence operon involved in protein synthesis (SSU ribosomal proteins)	4
**Resistance to antibiotics and toxic compounds**	
Beta-lactamase	1
Cobalt-zinc-cadmium resistance	4
Copper homeostasis	4
Mercuric reductase	2
Mercury resistance operon	1
Multidrug Resistance Efflux Pumps	2
Resistance to fluoroquinolones	4
Tetracycline resistance, ribosome protection type	2
Tetracycline resistance, ribosome protection type, too	2
**Total**	**1014**

Table S2. KEGG orthology (KO) categories of identified protein-coding genes in the *L. fermentum* JAC 231 genome.

CLASS - Category functional - Definition	Gene Count
**Cellular Processes**	**39**
**Cell growth and death**	**11**
Cell cycle - Caulobacter	10
Necroptosis	1
**Cell motility**	**3**
Cytoskeleton in muscle cells	1
Flagellar assembly	2
**Cellular community - prokaryotes**	**13**
Biofilm formation - Escherichia coli	1
Biofilm formation - Vibrio cholerae	2
Quorum sensing	10
**Transport and catabolism**	**12**
Efferocytosis	10
Peroxisome	2
**Environmental Information**	**89**
**Processing Membrane transport**	**49**
ABC transporters	31
Bacterial secretion system	8
Phosphotransferase system PTS	10
**Signal transduction**	**40**
HIF- 1 signaling pathway	6
MAPK signaling pathway - plant	2
Phosphatidylinositol signaling system	2
Two-component system	30
**Genetic Information Processing**	**156**
**Folding, sorting and degradation**	**27**
Protein export	10
RNA degradation	9
Sulfur relay system	8
**Replication and repair**	**66**
Base excision repair	9
DNA replication	15
Homologous recombination	19
Mismatch repair	16
Nucleotide excision repair	7
**Transcription**	**5**
RNA polymerase	5
**Translation**	**58**
Aminoacyl-tRNA biosynthesis	4
Ribosome	53
Ribosome biogenesis in eukaryotes	1
**Human Diseases**	**63**
**Infectious disease: viral**	**1**

Human papillomavirus infection	1
**Cancer: overview**	**11**
Central carbon metabolism in cancer	7
MicroRNAs in cancer	1
Pathways in cancer	1
Proteoglycans in cancer	1
Viral carcinogenesis	1
**Cancer: specific types**	**1**
Renal cell carcinoma	1
**Cardiovascular disease**	**9**
Diabetic cardiomyopathy	6
Fluid shear stress and atherosclerosis	2
Lipid and atherosclerosis	1
**Drug resistance: antimicrobial**	**15**
beta-Lactam resistance	3
Cationic antimicrobial peptide CAMP resistance	7
Vancomycin resistance	5
**Drug resistance: antineoplastic**	**5**
Antifolate resistance	4
Platinum drug resistance	1
**Endocrine and metabolic disease**	**4**
Cushing syndrome	1
Insulin resistance	1
Type I diabetes mellitus	1
Type II diabetes mellitus	1
**Infectious disease: bacterial**	**13**
Legionellosis	2
Pathogenic Escherichia coli infection	1
Salmonella infection	2
Staphylococcus aureus infection	5
Tuberculosis	3
**Infectious disease: parasitic**	**2**
Amoebiasis Immune disease	2
**Neurodegenerative disease**	**2**
Alzheimer disease	1
Parkinson disease	1
**Metabolism**	**1435**
**Amino acid metabolism**	**112**
Alanine, aspartate and glutamate metabolism	17
Arginine and proline metabolism	6
Arginine biosynthesis	12
Cysteine and methionine metabolism	21
Glycine, serine and threonine metabolism	11
Histidine metabolism	6
Lysine biosynthesis	14
Lysine degradation	3
Phenylalanine metabolism	2

Phenylalanine, tyrosine and tryptophan biosynthesis	5
Tryptophan metabolism	2
Tyrosine metabolism	6
Valine, leucine and isoleucine biosynthesis	2
Valine, leucine and isoleucine degradation	5
**Biosynthesis of other secondary metabolites**	**24**
Acarbose and validamycin biosynthesis	1
Biosynthesis of various antibiotics; Including: Kanosamine biosynthesis, Aurachin biosynthesis, Bacilysin biosynthesis, Puromycin biosynthesis, Dapdiamides biosynthesis, Fosfomycin biosynthesis, Cremeomycin biosynthesis, Fumagillin biosynthesis, Pentalenolactone biosynthesis, Terpentecin biosynthesis, Roseoflavin biosynthesis, Cycloserine biosynthesis	1
Biosynthesis of various plant secondary metabolites; Including: Crocin biosynthesis, Ginsenoside biosynthesis, Saponin adjuvant biosynthesis, Cannabidiol biosynthesis, Mugineic acid biosynthesis, Pentagalloylglucose biosynthesis, Benzoxazinoid biosynthesis, Gramine biosynthesis, Coumarin biosynthesis, Furanocoumarin biosynthesis, Hordatine biosynthesis, Podophyllotoxin biosynthesis	1
Carbapenem biosynthesis	2
Glucosinolate biosynthesis	1
Monobactam biosynthesis	4
Neomycin, kanamycin and gentamicin biosynthesis	1
Novobiocin biosynthesis	2
Penicillin and cephalosporin biosynthesis	1
Prodigiosin biosynthesis	3
Streptomycin biosynthesis	6
Tropane, piperidine and pyridine alkaloid biosynthesis	1
**Carbohydrate metabolism**	**153**
Amino sugar and nucleotide sugar metabolism	21
Butanoate metabolism	11
C-5 Branched dibasic acid metabolism	2
Citrate cycle (TCA cycle)	6
Fructose and mannose metabolism	7
Galactose metabolism	15
Glycolysis / Gluconeogenesis	18
Glyoxylate and dicarboxylate metabolism	5
Inositol phosphate metabolism	2
Pentose and glucuronate interconversions	6
Pentose phosphate pathway	14
Propanoate metabolism	11
Pyruvate metabolism	24
Starch and sucrose metabolism	11
**Energy metabolism**	**58**
Carbon fixation in photosynthetic organisms	6
Carbon fixation pathways in prokaryotes	11
Methane metabolism	7
Nitrogen metabolism	8

Oxidative phosphorylation	13
Photosynthesis	8
Sulfur metabolism	5
**Global and overview maps**	**801**
2-Oxocarboxylic acid metabolism	14
Biosynthesis of amino acids	62
Biosynthesis of cofactors	70
Biosynthesis of nucleotide sugars	19
Carbon metabolism	39
Degradation of aromatic compounds	5
Fatty acid metabolism	12
Nucleotide metabolism	28
Biosynthesis of secondary metabolites	148
Metabolic pathways	328
Microbial metabolism in diverse environments	76
**Glycan biosynthesis and metabolism**	**43**
Glycosaminoglycan degradation	1
Glycosphingolipid biosynthesis - globo and isoglobo series	1
Lipopolysaccharide biosynthesis	1
O-Antigen nucleotide sugar biosynthesis	9
Other glycan degradation	1
Peptidoglycan biosynthesis	20
Teichoic acid biosynthesis	10
**Lipid metabolism**	**36**
Fatty acid biosynthesis	11
Fatty acid degradation	4
Glycerolipid metabolism	11
Glycerophospholipid metabolism	8
Sphingolipid metabolism	2
**Metabolism of cofactors and vitamins**	**76**
Biotin metabolism	5
Folate biosynthesis	12
Lipoic acid metabolism	5
Nicotinate and nicotinamide metabolism	6
One carbon pool by folate	9
Pantothenate and CoA biosynthesis	8
Porphyrin metabolism	1
Retinol metabolism	2
Riboflavin metabolism	8
Thiamine metabolism	10
Ubiquinone and other terpenoid-quinone biosynthesis	7
Vitamin B metabolism	3
**Metabolism of other amino acids**	**28**
Cyanoamino acid metabolism	2
D-Amino acid metabolism	10
Glutathione metabolism	7
Selenocompound metabolism	6

Taurine and hypotaurine metabolism	3
**Metabolism of terpenoids and polyketides**	**18**
Biosynthesis of ansamycins	1
Biosynthesis of vancomycin group antibiotics	1
Nonribosomal peptide structures	1
Polyketide sugar unit biosynthesis	3
Terpenoid backbone biosynthesis	11
Zeatin biosynthesis	1
**Nucleotide metabolism**	**61**
Purine metabolism	37
Pyrimidine metabolism	24
**Xenobiotics biodegradation and metabolism**	**25**
Aminobenzoate degradation	1
Benzoate degradation	4
Chloroalkane and chloroalkene degradation	3
Dioxin degradation	1
Drug metabolism - cytochrome P	2
Drug metabolism - other enzymes	8
Metabolism of xenobiotics by cytochrome P	2
Naphthalene degradation	3
Xylene degradation	1
**Organismal Systems**	**19**
**Aging**	**3**
Longevity regulating pathway - worm	3
**Digestive system**	**2**
Fat digestion and absorption	1
Mineral absorption	1
**Endocrine system**	**9**
Glucagon signaling pathway	5
PPAR signaling pathway	2
Prolactin signaling pathway	1
Thyroid hormone synthesis	1
**Environmental adaptation**	**2**
Plant-pathogen interaction	2
**Immune system**	**1**
NOD-like receptor signaling pathway	1
**Nervous system**	**2**
GABAergic synapse	1
Glutamatergic synapse	1
**Total**	**1801**

Table S3. Orthologous clusters among *L. fermentum* strains JAC 231, AGR 1485, ATCC 23271, CECT 5716, IFO 3956, and MCC 2760.

Slimmed GO	Name	Count of unique input accessions
**Biological process**		
GO:0008152	metabolic process	500
GO:0008150	biological_process	477
GO:0044237	cellular metabolic process	424
GO:0006807	nitrogen compound metabolic process	351
GO:0044238	primary metabolic process	296
GO:0009987	cellular process	260
GO:0046483	heterocycle metabolic process	229
GO:0006725	cellular aromatic compound metabolic process	208
GO:0043170	macromolecule metabolic process	179
GO:0006139	nucleobase-containing compound metabolic process	178
GO:0006082	organic acid metabolic process	152
GO:0006793	phosphorus metabolic process	124
GO:0016070	RNA metabolic process	110
GO:0006412	translation	96
GO:0065007	biological regulation	89
GO:0009117	nucleotide metabolic process	85
GO:0051186	cofactor metabolic process	81
GO:0006810	transport	62
GO:0051234	establishment of localization	62
GO:0050896	response to stimulus	57
GO:0005975	carbohydrate metabolic process	55
GO:0009116	nucleoside metabolic process	53
GO:0016043	cellular component organization	43
GO:0006259	DNA metabolic process	40
GO:0019538	protein metabolic process	40
GO:0006629	lipid metabolic process	38
GO:0006396	RNA processing	37
GO:0006811	ion transport	36
GO:0006518	peptide metabolic process	35
GO:0043603	cellular amide metabolic process	34
GO:0044255	cellular lipid metabolic process	34
GO:0006260	DNA replication	32
GO:0032502	developmental process	31
GO:0051179	localization	30
GO:0042254	ribosome biogenesis	26
GO:0043412	macromolecule modification	26
GO:0006281	DNA repair	25
GO:0032989	cellular component morphogenesis	22
GO:0006091	generation of precursor metabolites and energy	19
GO:0006865	amino acid transport	19
GO:0007154	cell communication	18
GO:0032196	transposition	17
GO:0006766	vitamin metabolic process	15

GO:0051704	multi-organism process	12
GO:0006081	cellular aldehyde metabolic process	11
GO:0008643	carbohydrate transport	11
GO:0015031	protein transport	11
GO:0006464	cellular protein modification process	9
GO:0042180	cellular ketone metabolic process	9
GO:0051189	prosthetic group metabolic process	9
GO:0006457	protein folding	8
GO:0006818	hydrogen transport	8
GO:0005976	polysaccharide metabolic process	7
GO:0044419	interspecies interaction between organisms	7
GO:0051641	cellular localization	7
GO:0065003	macromolecular complex assembly	7
GO:0006066	alcohol metabolic process	6
GO:0006996	organelle organization	6
GO:0007049	cell cycle	6
GO:0009225	nucleotide-sugar metabolic process	6
GO:0009292	genetic transfer	6
GO:0022411	cellular component disassembly	6
GO:0022607	cellular component assembly	6
GO:0043094	cellular metabolic compound salvage	5
GO:0043101	purine-containing compound salvage	5
GO:0045333	cellular respiration	5
GO:0051276	chromosome organization	5
GO:0051301	cell division	5
GO:0006304	DNA modification	4
GO:0006730	one-carbon metabolic process	4
GO:0015074	DNA integration	4
GO:0016032	viral process	4
GO:0000003	reproduction	3
GO:0006662	glycerol ether metabolic process	3
GO:0007059	chromosome segregation	3
GO:0015849	organic acid transport	3
GO:0046490	isopentenyl diphosphate metabolic process	3
GO:0046903	secretion	3
GO:0006113	fermentation	2
GO:0006354	DNA-templated transcription, elongation	2
GO:0008655	pyrimidine-containing compound salvage	2
GO:0017144	drug metabolic process	2
GO:0032392	DNA geometric change	2
GO:0042440	pigment metabolic process	2
GO:0046794	transport of virus	2
GO:0051180	vitamin transport	2
GO:0071555	cell wall organization	2
GO:0001906	cell killing	1
GO:0006508	proteolysis	1
GO:0007005	mitochondrion organization	1
GO:0009308	amine metabolic process	1
GO:0009372	quorum sensing	1
GO:0015931	nucleobase-containing compound transport	1

GO:0015976	carbon utilization	1
GO:0019748	secondary metabolic process	1
GO:0031640	killing of cells of other organism	1
GO:0034622	cellular macromolecular complex assembly	1
GO:0042445	hormone metabolic process	1
GO:0042620	poly(3-hydroxyalkanoate) metabolic process	1
GO:0042710	biofilm formation	1
GO:0045229	external encapsulating structure organization	1
GO:0045230	capsule organization	1
GO:0051181	cofactor transport	1
GO:0051258	protein polymerization	1
**Cellular component list**		
GO:0044464	cell part	31
GO:0016020	membrane	25
GO:0005622	intracellular	7
GO:0005575	cellular_component	2
GO:0005576	extracellular region	1
GO:0005634	nucleus	1
GO:0005783	endoplasmic reticulum	1
GO:0043226	organelle	1
GO:0043229	intracellular organelle	1
**Molecular function list**		
GO:0003674	molecular_function	55
GO:0005215	transporter activity	51
GO:0016787	hydrolase activity	44
GO:0043167	ion binding	35
GO:0016740	transferase activity	34
GO:0003676	nucleic acid binding	27
GO:0008233	peptidase activity	19
GO:0016491	oxidoreductase activity	19
GO:0000166	nucleotide binding	18
GO:0005488	binding	14
GO:0001882	nucleoside binding	10
GO:0048037	cofactor binding	9
GO:0004871	signal transducer activity	4
GO:0005515	protein binding	3
GO:0008289	lipid binding	3
GO:0005198	structural molecule activity	2
GO:0016829	lyase activity	1
GO:0016874	ligase activity	1
GO:0019213	deacetylase activity	1
GO:0043021	ribonucleoprotein complex binding	1
GO:0060090	binding, bridging	1

Table S4. Subsystems related to vitamin synthesis identified in the *L. fermentum* JAC 231 genome.

Subsystems	Gene count
**Biotin**	
Biotin biosynthesis	3
**Coenzyme A**	
Coenzyme A Biosynthesis	7
**Cofactors, Vitamins, Prosthetic Groups, Pigments - no subcategory**	
Thiamin biosynthesis	8
**Folate and pterines**	
5-FCL-like protein	12
Folate Biosynthesis	10
Folate biosynthesis cluster	7
Molybdenum cofactor biosynthesis	11
**Lipoic acid**	
Lipoic acid metabolism	1
**NAD and NADP**	**8**
NAD and NADP cofactor biosynthesis global	8
**Pyridoxine**	
Pyridoxin (Vitamin B6) Biosynthesis	6
Pyridoxin(Vitamin B6) Degradation Pathway	1
**Riboflavin, FMN, FAD**	
Flavodoxin	3
riboflavin to FAD	4
Riboflavin, FMN and FAD metabolism	9
Riboflavin, FMN and FAD metabolism in plants	12
**Tetrapyrroles**	**3**
Heme and Siroheme Biosynthesis	3
Total	105

Table S5. Genes from non-pathogenic families identified by the PlasmidFinder v2.1 tool for *L. fermentum* JAC 231.

	PathogenFind			
Probability of being a human pathogen	0,203			
Corresponding Pathogenic Families	0			
Corresponding Nonpathogenic Families	10			
Class	Lactobacillares Organisms	Protein function	Identity (%)	Protein ID
	Lactobacillus helveticus DPC 4571	Cation transport protein	98,5	ABX27776
		Purine-cytosine permease	98,89	ABX27803
		Sugar kinase	99,66	ABX27804
		Carbonate dehydratase	98,1	ABX27797
	Lactobacillus reuteri DSM 20016	Transposase, IS605 OrfB family	90,77	ABQ83922
	Lactobacillus casei ATCC 334	Recombinase Transposition	98.12	ABJ69435
	Lactobacillus brevis ATCC 367	Site-specific, DNA invertase pin-related protein	96,26	ABJ65378
	Lactobacillus reuteri JCM 1112	Transposition putative metal-binding protein	89,81	SACO24586
	Lactobacillus delbrueckii subsp. bulgaricus ATCC 11842	(probable CopZ copper chaperone)	97,33	ABJ58075
		Crp-like transcriptional regulator	97,69	ABJ58075

Table S6. Predicted CRISPR-Cas systems in the genome of *L. fermentum* JAC 231.

READ	Spacers	DR Length	DR Consensus	Direction	Potential Orientation	Cas	CRISPR Start	CRISPR End	Length	CAS Start	CAS End
gnl_Prokka_EPNMGAAP_4	1	46	CRT results	ND	ND	Cas3_0_I	409	525	117	33092	34474
gnl_Prokka_EPNMGAAP_4	1	29	CRT results	ND	ND	Cas3_0_I	10070	10159	90	33092	34474
gnl_Prokka_EPNMGAAP_1	3	53	CRT results	ND	ND	Cas3_0_I	1769	2055	287	13702	15018
